# All‐Solid‐State Thin Film μ‐Batteries for Microelectronics

**DOI:** 10.1002/advs.202100774

**Published:** 2021-08-05

**Authors:** Tian Wu, Wei Dai, Meilu Ke, Qing Huang, Li Lu

**Affiliations:** ^1^ Hubei Engineering Technology Research Center of Environmental Purification Materials Hubei University of Education Gaoxin Road 129 Wuhan 430205 P. R. China; ^2^ Department of Mechanical Engineering National University of Singapore Singapore 117575 Singapore; ^3^ National University of Singapore Chongqing Research Institute Chongqing 401123 R. P. China; ^4^ National University of Singapore Suzhou Research Institute Suzhou 215123 R. P. China

**Keywords:** deposition, electrochemical behavior, solid‐state battery, solid‐state electrolyte, thin film μ‐battery

## Abstract

Continuous advances in microelectronics and micro/nanoelectromechanical systems enable the use of microsized energy storage devices, namely solid‐state thin‐film μ‐batteries. Different from the current button batteries, the μ‐battery can directly be integrated on microchips forming a very compact “system on chip” since no liquid electrolyte is used in the μ‐battery. The all‐solid‐state battery (ASSB) that uses solid‐state electrolyte has become a research trend because of its high safety and increased capacity. The solid‐state thin‐film μ‐battery belongs to the family of ASSB but in a small format. However, a lot of scientific and technical issues and challenges are to be resolved before its real application, including the ionic conductivity of the solid‐state electrolyte, the electrical conductivity of the electrode, integration technologies, electrochemical‐induced strain, etc. To achieve this goal, understanding the processing of thin films and fundamentals of ion transfer in the solid‐state electrolytes and hence in the μ‐batteries becomes utmost important. This review therefore focuses on solid‐state ionics and provides inside of ion transportation in the solid state and effects of chemistry on electrochemical behaviors and proposes key technology for processing of the μ‐battery.

## Graphical abstract

μ‐battery with its thickness in micrometer has gained increased attention in recent years due to reduced power consumption of modern microelectronics, and micro/nanoelectromechanical systems. However, there still exist tremendous issues to really integrate the μ‐battery into those microdevices. This review concentrates on the principle and challenges of the development of the μ‐batteries.

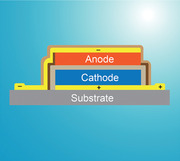

## 1 Introduction

**Figure 1:**
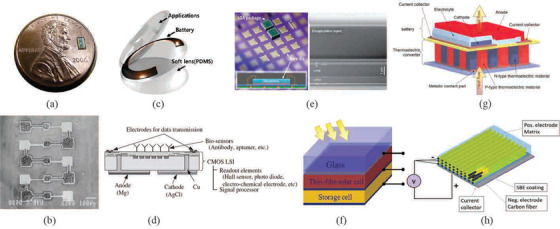
Examples of applications of thin film batteries. a) Image of an ATFB. Reproduced with permission.^[^ ^10^ ^]^ Copyright 2018, Springer Nature BV. b) Microcells of size 100 × 100 µm^2^ with nominal voltage 3.9V per cell. Reproduced with permission.^[^ ^11^ ^]^ Copyright 2002, IOP Publishing, Ltd. c) A pplication of the ATFB in smart lens. Reproduced with permission.^[^ ^17^ ^]^ Copyright 2018, Elsevier. d) On‐chip μ‐battery for biosensing and data transmission. Reproduced with permission.^[^ ^19^ ^]^ Copyright 2019, John Wiley & Sons. e) A large grid array package on Si wafer. Reproduced with permission.^[^ ^20^ ^]^ Copyright 2017, WileyVCH. f) ATFM integrated with a photovoltaic cell. Reproduced with permission.^[^ ^22^ ^]^ Copyright 2017, Elsevier. g) Illustration of a stand‐alone microsystem in which a P‐type thermoelectric device is integrated with an ATFB. Reproduced with permission.^[^ ^24^ ^]^ Copyright 2008, Elsevier. h) Fiber type of ATFB consisting of carbon fiber as the current collector as well as anode, polymer‐based electrolyte, and polymer/composite cathode. Reproduced with permission.^[^ ^25^ ^]^ Copyright 2019, Sage Publications.

The concept of thin‐film batteries or μ‐batteries have been proposed for a few decays.^[^ ^1^ ^]^ However it is a long and difficult match since the fabrication of the all‐solid‐state thin‐film μ‐batteries (ATFBs) relies on the development of solid electrolytes with reasonably high ionic conductivity and chemical and electrochemical stability. That is why it was also called thin‐film solid‐electrolyte batteries in the early days.^[^ ^2^, ^3^ ^]^ One of the early examples is Li/AgI thin‐film cell using simple but effective LiI as the electrolyte forming a Li/LiI/AgI all‐solid‐state thin‐film μ‐battery (ATFB) providing 2 V with a current density of over 100 μA cm^−2^.^[^ ^3^, ^4^ ^]^ Following Liang's works,^[^ ^3^, ^4^ ^]^ many ATFB were slowly developed using the different solid‐state electrolyte. For example, the cells were fabricated using borate glass B_2_O_3_‐*x*Li_2_O‐*y*Li_2_ SO_4,_^[^ ^5^ ^]^ Li_2_O‐V_2_O_5_‐SiO_2,_^[^ ^6^ ^]^ B_2_O_3_‐*x*Li_2_O,^[^ ^7^ ^]^ Li_3.6_Si_0.6_P_0.4_O_4,_^[^ ^8^ ^]^ Li_9_SiAlO_8,_^[^ ^9^ ^]^ etc. **Figure** **1**a reveals an example of a really small ATFB of dimension of 3.1 mm  × 1.7 mm × 95 µm.^[^ ^10^ ^]^ The cells can be connected in parallel and in series based on needs of different applications. Figure 1b shows scanning electron microscopy (SEM) of an integrated ATFB consisting of five microcells connected in serial giving a voltage of about 19.5 V.^[^ ^11^ ^]^

Due mainly to their low capacity, the ATFBs have been unable to find their practical applications until recently. With extraordinary advances in metal oxide semiconductor (CMOS) and micro/nano electromechanical systems (MEMS/NEMS), the consumption of energy of the electronic and micro/nanoelectromechanical devices becomes extremely low down to few mW.^[^ ^12^ ^]^ The importance of the ATFBs has been hence realized in ultralow power communications, wireless sensors, and detectors,^[^ ^13^ ^]^ MEMS/NEMS,^14^ medical devices, biosensors,^[^ ^15^ ^]^ and wearable devices.^[^ ^16^ ^]^ Due to micron size in thickness of the ATFB, it has been considered to be the potential candidates for microwearable devices. A smart lens (contact lens) as shown in Figure 1c is an innovative example where the ATFM is fabricated on a polyimide substrate‐based contact lens.^[^ ^17^ ^]^ Another example is on‐chip μ‐battery to provide energy for biosensing device such as an immunoreaction‐based disposable complementary CMOS biosensing chip (Figure 1d).^[^ ^18^, ^19^ ^]^ The μ‐battery can be deposited or printed on the CMOS chip. The biosensing chip consists of biosensors, wireless optical data transmission, and a chloride Mg–AgCl incomplete μ‐battery without the electrolyte. Since biocharacterization is usually carried out in an aqueous solution such as saline that contains 0.9% (w/v) NaCl or with additives of NaCl or NH_4_Cl, the aqueous solution for sensing can be used as the electrolyte. Figure 1e shows a typical example of an ATFB where an ATFB is directly fabricated on the Si wafer, which can be further integrated with microchips.^[^ ^20^ ^]^ Since the ATFBs are fabricated through thin‐film technologies, they can be directly integrated into microchips forming very compact self‐powered system‐on‐chip and/or laboratory‐on‐chip.^[^ ^15^ ^]^ When the self‐powered system‐on‐chip is further integrated with an energy harvesting system, this final system will be self‐retained.^[^ ^21^ ^]^ One of the immediate designs is to integrate a photovoltaic (PV) cell with the ATFB as schematically illustrated in Figure 1f.^[^ ^22^ ^]^ The electric energy that is harvested via the PV cell is stored in the ATFB through electrochemical conversion.^[^ ^23^ ^]^ Another example is the stand‐alone microsystems where an ATFB is integrated with a thermoelectric device Figure 1g.^[^ ^24^ ^]^ A small temperature difference over 3 °C is essential. In addition to the planar type of ATFBs, the fiber type of ATFBs opens another opportunity for wearable devices. Different from the planar type, the fiber type of ATFBs is made of polymers due to the need of flexibility.^[^ ^16^, ^25^ ^]^ Since a fiber of a few tenth micrometers in diameter is very flexible, it can easily be weaved into different forms for different usages. For the planar type of ATFBs, metals are used as the current collectors, while for the fiber type of ATFBs, flexible carbon fibers are used as the current collector and at the same time it plays the role as the negative electrode as well (Figure 1h).^[^ ^25^ ^]^

This review will provide fundamentals of the ATFBs, state‐of‐the‐art of ATFBs, challenges, and their potential applications particularly in the microelectronic era. The review will only focus on the ATFBs from inorganic materials.

## 2 Fundamentals of All‐Solid‐State Thin‐Film μ‐Batteries

**Figure 2:**
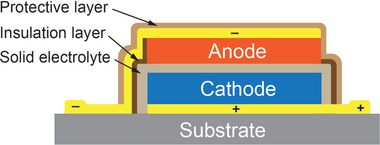
Schematic illustration of an ATFB consisting of a cathode, an anode and a solid‐state electrolyte.

A battery is an electrochemical galvanotactic cell that physically consists of a positive electrode, a negative electrode, and an electrolyte. The role of the two electrodes is to generate and consume electrons whereas the electrolyte only allows flow of ions, namely an electron insulator.^[^ ^26^ ^]^ ATFBs have no exception. As illustrated in **Figure** **2**, an ATFB is also made of three essential components but in a thin film format. Because of the same physical configuration of both types of batteries, the same thermodynamic formulation results in the same theoretical open‐circuit voltage (OCV) that is a measure of the difference in free energy, Δ*F* ^o^, between the two electrodes as shown in Equation (1),^[^ ^27^ ^]^

(1) $$E^{o}=\frac{-\Delta F^{o}}{nF}\;$$

where *n* is the number of electrons and *F* is the Faraday constant.

The migration of metal ions, such as Li^+^, is through interstitial hopping mechanisms that can be represented by Frenkel defect reaction

(2) $$M_{M}^{\times }+V_{i}^{\times }\to M_{i}^{•}+V_{M}^{\prime }$$

where $M_{M}^{\times }$ represents a metal ion staying its position interstitially with an interstitial vacant site, $V_{i}^{\times }$. When the metal ion moves from its original site to a new interstitial site, its original site is hence negatively charged, $V_{M}^{\prime }$, whereas the newly occupied interstitial site is positively charged, $M_{i}^{•}$. It is therefore clear that to have good migration of Li^+^ ions, the electrolytes should have sufficient charge carriers since the ionic conductivity, *σ*, is proportional to the density of the charge carrier, *c_i_*, through

(3) $$\sigma \;=q_{i}\;\mu_{i}c_{i}$$

where *q_i_* is the charge of the ions and μ_*i*_ is the mobility of the ions which can be calculated by Nernst‐Einstein equitation, and

(4) $$\mu_{i}=D_{i}\;q_{i}/\kappa T$$

where *κ* is the Boltzmann constant and *T* is the absolute temperature. At the same time, there should be enough vacant interstitial sites for Li^+^ ions to hop. Since ion hopping is driven by thermal activation, from Fick's second law, and Nernst‐Einstein equitation, the metal ion, in this case Li^+^ conductivity, *σ* can therefore be obtained

(5) $$\sigma T\;=\sigma_{0}\;\exp\left(-E_{a}/kT\right)$$

where *σ*_0_ is the preexponential constant and *E*_a_ is the activation energy.

Accompanied by ion migration through the electrolyte, the charge transfer takes place at the interface between the electrolyte and the electrode. As such the electrode not only needs to have good ionic conductivity but also good electronic conductivity to transfer electrons. Unfortunately, the bare electrode materials have very poor electronic conductivity. To increase the electronic conductivity in the electrode of a bulk battery, a certain amount of conductive materials such as carbon and carbon derives should be incorporated with the electrode materials, and sometimes the electrode particles are even coated with carbon layers in order to provide reasonably good electronic conductivity.

**Figure 3:**
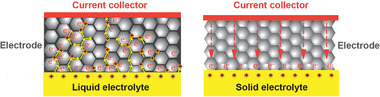
Comparison of ions and electrons migration in a) a bulk Li‐ion battery and b) an ATFB.

However, since the process of a thin‐film fabrication is entirely different from the fabrication of the bulk batteries, in addition to the prerequisite of high ion storage (such as Li ion storage for Li‐ion batteries), both positive and negative electrodes should have reasonably good ionic as well as electronic conductivity in order to reduce cell resistance. **Figure** **3** compares two different configurations. For the bulk Li‐ion battery, the electrode is made of the active material and the conductive carbon‐black (Figure 3a) where the Li ions migrate through the liquid electrolyte that penetrates through the binder and conductive carbon‐black. Therefore, the charge transfer can be realized at the interface between the carbon and the electrolyte resulting in very low cell resistance and good kinetics. On the other hand, the ATFB is fabricated mostly through thin film deposition technologies in which it is hard and almost impossible to incorporate electronic conductive materials in the electrode. Furthermore, since the Li ions migrate through diffusion from the interface between the electrolyte and the electrode, the charge transfer in the ATFBs is entirely dependent on the electronic conductivity of the electrode itself as illustrated in Figure 3b, whereas most electrode materials such as LiFePO_4_, LiCoO_2_, LiNi_1/3_Mn_1/3_Co_1/3_O_2_ etc. have extremely poor electronic conductivity. Although both types of the battery share the same thermodynamic description as described in Equation (1) at zero current, there is a large voltage polarization during charge and discharge in the ATFB when the current is not equal to zero since the cell resistance of the ATFB is few tenth order of magnitude of that of the bulk ones. To reduce the cell resistance, the thickness of the electrodes has to be only about a few micrometers.

Furthermore, almost all available solid‐state electrolytes have lower ionic conductivities between 10^−7^ S cm^−1^ and 10^−3^ cm^−1^ in comparison with the liquid organic electrolyte of 10^−2^ S cm^−1^. To reduce ion migration resistance, the electrolyte should be as thin as possible usually in the range of about a micron. This is why it is called “μ‐battery” in the sense of thickness.

## 3 Solid‐State Electrolytes

One of the key challenges in developing the ATFBs is searching for suitable solid‐state electrolytes. The suitable materials for electrolyte must fulfill the following three essential requirements in the material level: a) high ionic conductivity preferably higher than 10^−5^ S cm^−1^ to provide easy migration of charge carriers, b) low electronic conductivity at least less than 10^−9^ S cm^−1^ to ensure neglectable current leakage and hence long shelf‐life, and c) high rigidity to have mechanical integrity. As the thin‐film solid‐state electrolyte, further to the essential requirements from the material level, the electrolyte materials must also be suitable for thin film deposition processes, the electrolytes should be preferably deposited at relatively low temperature to avoid inter‐diffusion between the electrolyte and the electrode, the thin film solid‐state electrolytes should be absolutely pin‐holes‐free with smooth surfaces, and finally less grain boundaries and tight grains are also essential. Because of so many constraints, developing and searching the suitable solid‐state ionic conductors for thin‐film electrolyte are slow. In a broad spectrum, the solid‐state electrolytes can be categorized into two categories, namely amorphous‐based solid‐state electrolytes and crystalline‐based ones. The amorphous‐based solid‐state electrolytes typically have lower ionic conductivities in comparison with their count part of the crystalline ones, but the amorphous‐based electrolytes may provide uniform ion migration pathway and much dense microstructure. Due to crystalline in nature, the crystalline electrolyte behaves some differences in ionic and electronic conductivities between grains and grain boundaries. Very often, microvoids can be found between grains.

### 3.1 Amorphous Electrolytes

#### 3.1.1 Lithium Phosphate and Its Derives

**Figure 4:**
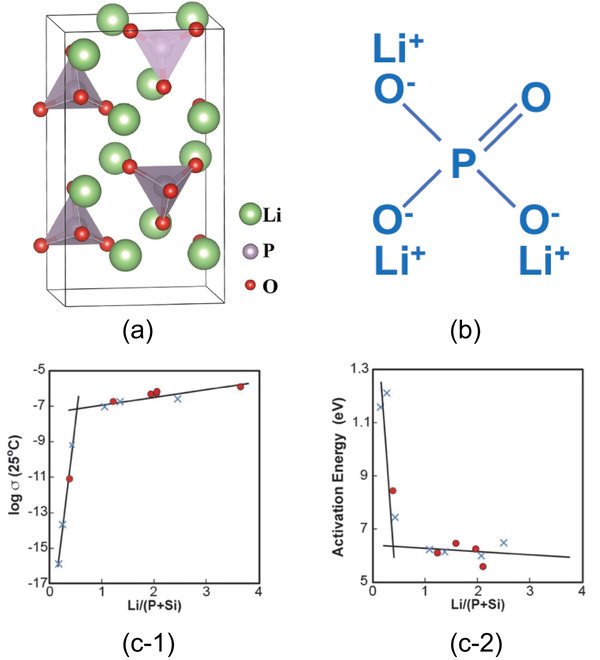
Lithium orthophosphate, Li_3_PO_4_, a) lattice structure. Reproduced with permission.^[^ ^34^ ^]^ Copyright 2017, AIP Publishing. b) Chemical bonds, and c‐1) ionic conductivity and c‐2) activation energy of (1‐*x*)Li_4_SiO_4_ *x* Li_3_PO_4_. Reproduced with permission.^[^ ^35^ ^]^ Copyright 1992, Elsevier.

Lithium phosphorus oxynitride that is also called as LiPON (Li*_x_* PO*_y_* N*_z_*, where 2.6b < *x* < 3.5, 1.9 < *y* < 3.8 and 0.1 < *z* < 1.3), is an old but very successful amorphous type of solid electrolyte.^[^ ^28^, ^29^, ^30^, ^31^, ^32^ ^]^ The origin of LiPON is lithium phosphate (LiPO) in the form Li_4_P_2_O_7_, Li_3_PO_4_, and LiPO_3_.^[^ ^33^ ^]^ The typical example is Li_3_PO_4_, where P^5+^ and O^2−^ form isolated (PO_4_)^3 −^ tetrahedra in which a P^5+^ stays inside (**Figure** **4**a).^[^ ^34^ ^]^ The molecules of lithium orthophosphate are coordinated through doubly‐coordinated oxygen (Figure 4b).

LiPO in the bulk form can be synthesized easily through solid‐state reaction, melt‐quench, and welt chemical method. There is a large difference in congruent melting temperature for different compositions, which brings some choices of selection of different composition. The melting temperatures of lithium orthophosphate, lithium pyrophosphate, and lithium metaphosphate are 1225 °C, 885 °C, and 688 °C, respectively.^[^ ^34^ ^]^ By assuming direct hoping of Li^+^ from one equilibrium site to another in the lithium orthophosphate, Nudged‐elastic‐band (NEB) calculation reveals Li^+^ ions migration path if the distance of the migration to the neighbor vacancy site is shorter than 3.5 Å after overcoming a barrier energy of 0.58 eV calculated by density function theory (DFT).^[^ ^34^ ^]^

Since LiPO has a low ionic conductivity, solid solution has therefore been introduced. Introduction of Li_4_SiO_4_ into LiPO to form (1‐*x*)Li_4_SiO_4_ *x* Li_3_PO_4_ showed an increase in Li^+^ conductivity about one to two order of magnitude high than that of the pristine LiPO.^[^ ^35^ ^]^ Figure 4c‐1 reveals clearly a sharp increase in Li^+^ conductivity with a slight increase in Li/(P+Si) ratio followed by a gentle increase with a further increase in Li/(P+Si). Since a change in Li concentration does not drastically alter the preexponential constant (Equation 5), the sharp increase is hence ascribed by the activation energy as also clearly presented in Figure 4c‐2.

**Figure 5:**
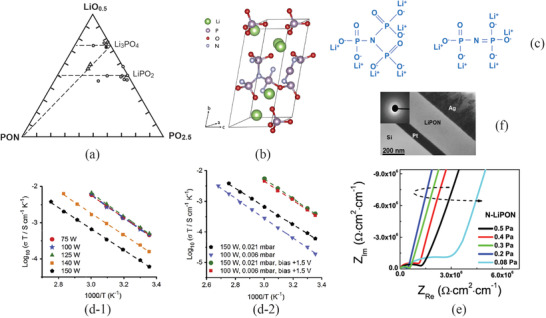
a) LiO_0.5_‐PON‐PO_2.5_ ternary phase diagram with a dashed line showing changes of N concentration from bare Li_3_PO_4_. Reproduced with permission.^[^ ^36^ ^]^ Copyright 1969, Elsevier. b) The unit cell of LiPON. Reproduced with permission.^[^ ^37^ ^]^ Copyright 2016, Elsevier. c) Crosslinked LiPON through N triply bond. d) Arrhenius plots of the ionic conductivities at different deposition parameters: d‐1) influence of power density of rf‐sputtering, and d‐2) effect of bias potential. Reproduced with permission.^[^ ^48^ ^]^ Copyright 2018, Elsevier. e) Impedances of LiPON thin films at a different partial pressure of N_2_ gas. Reproduced with permission.^[^ ^49^ ^]^ Copyright 2018, Elsevier. f) The cross section of LiPON on Pt sublayer deposited using IBS technique showing extremely smooth surface. Reproduced with permission.^[^ ^52^ ^]^ Copyright 2015, Elsevier.

Further detailed study with the incorporation of more SiO_2_, namely lowering Li concentration discovered the increase in the ionic conductivity by a factor of three, leading to a very important conclusion that the structure of the amorphous LiPO consisting of monomers and linear chain polymers changed to a crosslinked structure due to presence of SiO_2_.^[^ ^35^ ^]^ This finding led to further development in LiPON thin films. The composition of LiPON varies from its theoretical value Li_3_PO_3_N_2/3_ because of the difficulty of incorporation of N at high temperature. **Figure** **5**a reveals the ternary phase diagram of LiO_0.5_–PON–PO_2.5_^[^ ^36^ ^]^ in which the compositional changes from LiPO to LiPON, and its unit cell is schematically shown in Figure 5b. Similar to the incorporation of SiO_2_, introducing N in the LiPO causes the formation of the N triply bonds that crosslinks monomers together facilitating Li^+^ hopping.^[^ ^37^ ^]^ Based on Munoz,^[^ ^38^ ^]^ the crosslinked LiPON possesses three kinds of bonds, namely doubly‐ and triply coordinated nitrogen, and bridging oxygen. The illustration of N triply coordinated bond is shown in Figure 5c. The most important discovery of LiPON is not only on its high ionic conductivity of about 10^−6^ S cm^−1^ but also extremely low electronic conductivity of 10^−14^ S cm^−1^. Its chemical stability is much better than LiPO against metallic Li because (1‐*x*)Li_4_SiO_4_ *x* Li_3_PO_4_ is unstable when it contacts with metallic Li from the direct observation.^[^ ^37^, ^38^ ^]^

For a certain period of time, LiPON has been commonly considered to be stable when in contact with metallic Li. However, recent experimental and theoretical investigations show otherwise. When it contacts with metallic Li, LiPON would be reduced, which is a thermodynamically favorable process.^[^ ^37^, ^39^ ^]^ Experimental study discovered that the triply nitrogen‐coordinated LiPON, in specific Li_6_P_3_O_9_N, broken up into Li_3_PO_4_ and Li_4_P_2_O_7_ small units and other species such as Li_3_N and Li_2_O.^[^ ^40^ ^]^

The techniques of deposition of the LiPON thin film are limited because it is difficult to incorporate N atoms into the deposited thin film. The most popular technique is in situ rf‐sputtering growth,^[^ ^41^, ^42^, ^43^ ^]^ in which the in situ rf‐sputtering is carried out using Li_3_PO_4_ as the target in a N_2_ atmosphere.^[^ ^44^, ^45^, ^46^ ^]^ During the deposition, N_2_ gas is ionized and the ionized N ions then react with Li–P–O species forming in situ LiPON on the substrate. Deposition of LiPON is difficult since it should be deposited at low temperature in order to achieve an amorphous structure. At a low deposition power density and low temperature, the surface with an amorphous structure reveals featureless. The growth rate of LiPON deposition is extremely low, typically in about a 100 nm range per hour depending on the deposition temperature. For example, the growth rate of the LiPON thin film was as lower as 175 nm h^−1^ at 130 °C.^[^ ^47^ ^]^ Deposition power density is usually low about 0.8–2 W cm^−2^ because high deposition power may cause granular fracture of the LiPO targets and droplets of the target on the substrate. It was also noted that ionic conductivity was independent of the power of rf‐deposition at low power density range. However, as shown in Figure 5d‐1, when the power density of the rf‐sputtering is higher than the certain regime, the ionic conductivity quickly drops with an increase in power density of deposition.^[^ ^48^ ^]^ More detailed study by Jouybari et al.^[^ ^48^ ^]^ showed that substrate floating potential increased from a negative value at a low rf‐sputtering power to a positive value at a higher deposition power. The change in substrate floating potential in turn results in the redistribution of the electric field between the anode and cathode, and hence composition of the LiPON thin film. To compensate the increase in the substrate floating potential, an effective way is to introduce a bias. As demonstrated in Figure 5d‐2, at the same rf‐sputtering power the ionic conductivity can be increased with a bias as low as +1.5 V to compensate the increase in the substrate floating potential.

The partial pressure of the N_2_ gas plays an important role since it determines the amount N that can be incorporated in the LiPON, leading to variation of the impedance.^[^ ^49^, ^50^ ^]^ Figure 5e shows the change in impedance with the partial pressure of N_2_ gas.^[^ ^49^ ^]^ The impedance value of the LiPON thin film decreases with an increase in N_2_ partial pressure. The changes in the impedance can be ascribed to the nature of the floating potential again. The change in the floating potential during a deposition will alter the voltage of the target leading to the compositional change on the surface of the target. It was noted that when the N_2_ partial pressure was increased from 0.001 to 0.05 mbar, it caused a drop of about 10 V in substrate potential.^[^ ^48^ ^]^ However, there is a slight increase in impedance at higher pressures. One note being worth mentioning is that the Li loss during preparation of the LiPO target and deposition is critical. Direct sintering of the LiPO target will cause evaporation of Li causing lower Li concentration. Therefore, compositional compensation must be considered. One of the examples is to compensate Li loss through incorporation a substantial amount of Li_2_O in Li_3_PO_4_ in an 1:1 mole ratio before sintering.^[^ ^51^ ^]^

Ion beam sputtering (IBS) is another sophisticated deposition method that can be used to deposit ultrathin high‐quality LiPON.^[^ ^52^ ^]^ The IBS provides significant advantages in low‐temperature deposition by separation of substrate from the sputter plasma, resulting in negligible thermal impact on the substrate and hence low thermal stresses and interfacial reactions between the deposited LiPON and its sublayer. Figure 5f reveals the cross section of a LiPON thin film of about 205 nm on Pt sublayer. The LiPON thin film has a smooth surface and defects free.^[^ ^52^ ^]^ It has been claimed that IBS can provide a smooth surface with a roughness less than 1 nm.^[^ ^53^ ^]^

#### 3.1.2 Li–V–Si–O Solid Electrolyte

**Figure 6:**
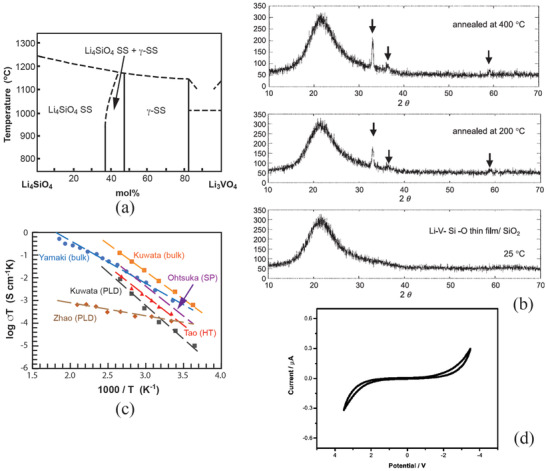
a) Li_4_SiO_4_–Li_3_VO_4_ partial phase diagram. Reproduced with permission.^[^ ^55^ ^]^ Copyright 1984, Elsevier. b) Structural evolutions at PLD deposition at 25 °C and after annealing at 200 °C and 400 °C, respectively. Reproduced with permission.^[^ ^61^ ^]^ Copyright 2004, Elsevier. c) Temperature dependence of LVSO electrolytes prepared by bulk, rf‐sputtering, PLD and hydrothermal processes (Kuwata (PLD and Li_3.4_V_0.6_Si_0.4_O_4_, bulk),^[^ ^60^ ^]^ Ohtsuka (Li_3.4_V_0.6_Si_0.4_O_4_, SP),^[^ ^64^ ^]^ Zhao (Li_6.16_V_0.61_Si_0.39_O_5.36_, PLD at 300 °C),^[^ ^65^ ^]^ Tao (Li_3.4_V_0.6_Si_0.4_O_4,_ HT),^[^ ^63^ ^]^ and Yamaka (Li_3.4_V_0.6_Si_0.4_O_4_, bulk),^[^ ^66^ ^]^ and d) cyclic voltammogram of LVSO thin film at a scan rate of 10 mV s^−1^. Reproduced with permission.^[^ ^65^ ^]^ Copyright 2003, Elsevier.

Li_2_O–V_2_O_5_–SiO_2_ (LVSO) is another kind of solid‐state electrolyte often used in the amorphous state. The composition of LVSO can generally be presented as Li_3+_ *_x_* V*_x_* Si_1‐_ *_x_* O_4_.^[^ ^54^ ^]^ **Figure** **6**a shows Li_4_SiO_4_–Li_3_VO_4_ phase diagram in which there are two single phase zones, Li_4_SiO_4_ solid solution (SS) and a zone with a *γ*‐Li_3_PO structure.^[^ ^55^ ^]^ Li_4_SiO_4_ has a monoclinic structure with a space group *P*2_1_/*m* containing [SiO_4_]‐tetrahedra that is connected by [LiO*_n_*]‐polyhedra.^[^ ^56^ ^]^ In between, there is a two‐phase zone, namely Li_4_SiO_4_ SS and *γ*‐phase SS. The *γ*‐phase SS possesses a structure similar to Li_4_SiO_4_ but with an orthorhombic symmetry with a space group *Pnma*.^[^ ^57^ ^]^ Li_3_VO_4_ can largely solid‐solute into Li_4_SiO_4_ up to 37 mol%. Between 37 and 46 mol%, Li_4_SiO_4_ SS and *γ*‐phase SS coexist. The single *γ*‐phase SS ranges from 46 to 82 mol%. A typical composition of LVSO is Li_3.4_ V_0.4_Si_0.6_O_4_.

Its ionic conductivity typically ranges from 10^−7^ to 10^−6^ S cm^−1^ with an activation energy of about 0.5 eV depending type of deposition technique used. The electronic conductivity of LVSO prepared by PLD is as low as about 10^−13^ S cm^−1^ at 25 °C with Li^+^ transport number almost unity whereas that prepared by rf‐sputtering is about 10^−10^ S cm^−1^. The LVSO film prepared by rf‐sputtering has higher conductivity than those prepared by other methods such as by the pulsed laser deposition (PLD).^[^ ^6^, ^58^, ^59^, ^60^ ^]^ It was noted that the film with an amorphous structure is more sensitive to ambient condition and easy to reaction of H_2_O and CO_2_ leading to the formation of LiOH and Li_2_CO_3_. As a consequence, ionic conductivity is lowered.^[^ ^58^ ^]^ Deposition at room temperature forms an amorphous structure that can be partially crystallized at an elevated temperature. The experimental observation reveals start of crystallization at as low as 200 °C (Figure 6b).^[^ ^61^ ^]^ However, very high conductivity of the bulk 40Li_4_SiO_4_‐60Li_3_VO_4_ of about 10^−5^ S cm^−1^ at 20 °C was also reported.^[^ ^55^ ^]^ The high ionic conductivity might be associated with different valence state of vanadium.

The LVSO was also synthesized by solvothermal process showing a crystalline structure having a relative higher ionic conductivity of 8.92 × 10^−7^ S cm^−1^ with an activation energy of 0.49 eV at 25 °C.^[^ ^62^, ^63^ ^]^ Figure 6c reveals the ionic conductivities of the LSVO thin films prepared by PLD, rf‐sputtering (SP), hydrothermal (HT). The ionic conductivity of the bulk LSVO shows slightly higher. The thin films prepared by rf‐sputtering demonstrate an ionic conductivity of about one order of magnitude higher than that prepared by PLD.

Although LVSO has many advantages with reasonably high ionic conductivity, high ion transference number, and easy processing, its main drawback is its electrochemical instability due to change of the valance of V^5+^. Figure 6d shows cyclic voltammogram (CV) of LVSO with a nominal composition Li_6.16_V_0.61_Si_0.39_O_5.36_. The LVSO film that was deposited at 300 °C by PLD was still shown amorphous in nature but with only small trace of humpers. The stable potential window is only 3.5 V much narrow than that of LiPON.^[^ ^65^ ^]^ Even though, LVSO is still considered to be attractive since it has no reaction with metallic Li.^[^ ^63^ ^]^

### 3.2 Crystalline‐Structured Electrolytes

Compared with amorphous type of electrolytes, there are many choices in crystalline‐structured electrolytes, such as garnet‐,^[^ ^67^, ^68^, ^69^ ^]^ LISICON‐,^[^ ^70^ ^]^ NASICON‐,^[^ ^74^, ^140^ ^]^ perovskite‐type etc.

#### 3.2.1 Garnet‐Type of Electrolyte

**Figure 7:**
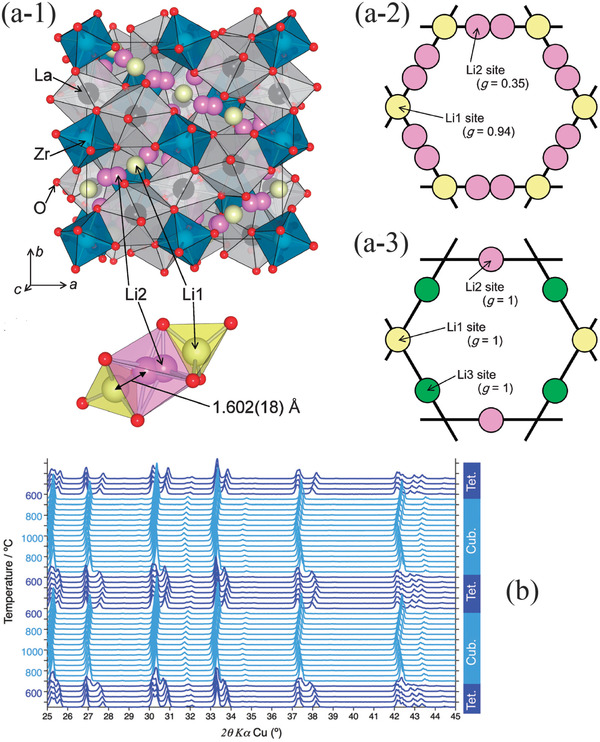
a‐1) Illustration of the crystal structure of Li_7_La_3_Zr_2_O_12_, arrangement of Li ions in a‐2) a cubic‐structured LLZO and in a‐3) a tetragonal‐structured LLZO. Reproduced with permission.^[^ ^80^ ^]^ Copyright 2011, The Chemical Society of Japan. b) Structural evolution of LLZO revealing changes from a tetragonal to a cubic during heating and from the cubic to the tetragonal during cooling. Reproduced with permission.^[^ ^82^ ^]^ Copyright 2014, Royal Society Chemistry.

Garnet‐type of solid electrolyte can be written in the general formula A_3_B_2_X_3_O_12_ where A, B, and X sites are eightfold coordinated, sixfold, and fourfold coordinated, respectively. Li7 (7 Li ions per formula) garnet solid electrolyte with the typical chemical composition Li_7_La_3_Zr_2_O_12_ (LLZO) was developed in 2007.^[^ ^67^ ^]^ **Figure** **7**a‐1 presents the structure of LLZO that is composed of dodecahedral LaO_8_ and octahedral ZrO_6_ where La, Zr and O ions occupy 24c, 16a and 96h sites, respectively.^[^ ^80^ ^]^ The Li ions locate at two sites, namely 24d (Li1) and 96h (Li2). For Li2, there are two equivalent sites in the distorted octahedral (lower section of Figure 7a‐1).^[^ ^80^ ^]^

For a bare LLZO, it has a tetragonal crystal structure at the room temperature and transforms to a cubic structure at about 650 °C. This transformation is reversible, and the high‐temperature cubic phase will transform back to a tetragonal structure when it is cooled to about 600 °C as shown in Figure 7b.^[^ ^82^ ^]^

If only Li ions are concerned, for the cubic LLZO, a complete loop structure forms by Li1 and Li2 in which Li1 site is shared by two loops (Figure 7a‐2), whereas for the tetragonal structure one tetragonal site and two octahedral sites are fully occupied by Li1, Li2, and Li3, respectively (Figure 7a‐3).^[^ ^80^ ^]^ The Li ions in the cubic LLZO are ordered in three different sites, whereas the diffusion pathways in the tetragonal LLZO are anisotropic.^[^ ^87^ ^]^

Since cubic‐structured LLZO possesses a short migration distance and sufficient Li^+^ vacancies, the ionic conductivity of the cubic‐structured LLZO is two order of magnitude higher than that of the tetragonal‐structured one.^[^ ^80^, ^81^, ^82^ ^]^ However, unfortunately, the cubic‐structured LLZO is unstable at room temperature. To stabilize the high‐temperature cubic phase at room temperature, various doping technologies have been used. One of the typical examples is Al doping to stabilize the high‐temperature cubic phase to room temperature through formation of vacancies at Li sites,^[^ ^67^, ^83^, ^84^, ^85^, ^86^ ^]^

(6) $${\mathrm{Al}}_{\mathrm{Al}}^{*}={\mathrm{Al}}_{\mathrm{Li}}^{••}\;+2V_{\mathrm{Li}}^{\prime }$$

**Figure 8:**
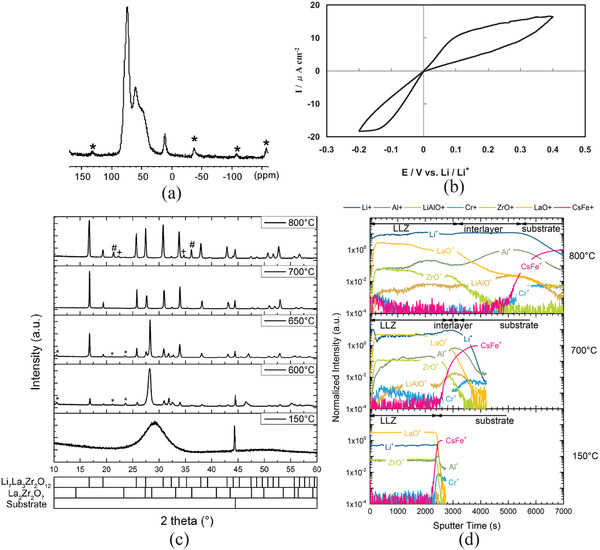
Properties of garnet‐structured LLZO: a) Al MAS NMR showing a typical signal of octahedral Al at 11.8 ppm. Reproduced with permission.^[^ ^87^ ^]^ Copyright 2011, American Chemical Society. b) Cyclic voltammetry of Li/LLZO/Li at a voltage swept rate of 10 mV min^−1^. Reproduced with permission.^[^ ^97^ ^]^ Copyright 2010, IOP Publishing, Ltd. c) Structural evolution of Li_6.6_La_3_Zr_1.6_Ta_0.4_O_12_ deposited at a different substrate temperature in which only amorphous phase can be seen at 150 °C and cubic‐structured LLZO forms only at 750 °C,  and d) depth profile of chemical composition measured by ToF‐SIMS. c,d) Reproduced withpermissioin.^[^ ^102^ ^]^ Copyright 2016, Elsevier.

Al magic‐angle spinning nuclear magnetic resonance (MAS NMR) spectroscopy provided information of octahedral Al at LaAlO_3_ which is indicated by the MAS NMR peaks at 11.8, 81, and 68 ppm (**Figure** **8**a).^[^ ^87^ ^]^ The substitution of Li^+^ by heterovalent Al^3+^ decreases the Li site occupancy and hence increases Li vacancies for Li ions easy migration. Another substitution of Li^+^ that was often used is Ga^3+^. Since the ionic size of Ga^3+^ is bigger than that of Al^3+^, it is believed that Ga^3+^ occupies Li1 tetrahedral site lowering occupancy fraction.^[^ ^88^ ^]^ Although substitution of Li by Al^3+^ and/or Ga^3+^ for example can stabilize its cubic structure, the substituted ions will most likely block Li ions’ migration. Therefore, substitutions on other sites instead of Li ion sites have also been studied. For example, tetravalent Zr^4+^ at 16*a* site is substituted by pentavalent, tetravalent and trivalent Ta^5+^, Nb^5+^, Ce^4+^, Cr^3+ [^ ^89^ ^]–[^ ^92^ ^]^ leading to formation of Li^+^ vacancies as

(7) $${M^{5+}}_{M}^{*}={M^{5+}}_{Zr^{4+}}^{•}\;+V_{\mathrm{Li}}^{\prime }$$

or leading to increased Li^+^ concentration as

(8) $${M^{3+}}_{M}^{*}={M^{3+}}_{Zr^{4+}}^{\prime }+{\mathrm{Li}}_{i}^{•}$$

The same expression is also held for substitution of trivalent La^3+^ at 24c site by divalent Ca^2+^, Ba^2+^, Mg^2+^, Sr^2+^ and tetravalent Ce^4+^.^[^ ^93^, ^94^, ^95^ ^]^

Furthermore, LLZO also demonstrates high electrochemical stability within a wide range of potential.^[^ ^67^, ^87^, ^96^ ^]^ Figure 8b shows the chronopotentiogram of Li/LLZO/Li cell and no reduction was discovered when it was directly contacted to metallic Li.^[^ ^97^ ^]^

Compared to LiPON, there are vast choices for the preparation of the garnet‐structured thin‐film electrolytes since the composition of the as‐deposited film can be directly correlated to the chemical composition of the targets and chemicals used, including sputtering,^[^ ^98^, ^99^, ^100^, ^101^, ^102^ ^]^ PLD,^[^ ^103^, ^104^, ^105^ ^]^ chemical vapor deposition (CVD),^[^ ^106^ ^]^ and chemical solution method.^[^ ^107^, ^108^, ^109^, ^110^ ^]^ Although there are many methodologies to grow crystalline garnet thin film, high‐temperature processing is essential to ensure better crystallinity and hence high ionic conductivity.

Room temperature rf‐sputtering of LLZO can only obtain an amorphous structure that has the ionic conductivity as low as 10^−7^ S cm^−1^.^[^ ^98^ ^]^ To have a crystalline LLZO, the rf‐sputtering is usually carried out at least above 600 °C, and often postannealing is also needed to achieve high crystallinity and density. Structural evolution of LLZO of target composition Li_6.6_La_3_Zr_1.6_Ta_0.4_O_12_ which was deposited at different substrate temperatures is shown in Figure 8c.^[^ ^102^ ^]^ At 600 °C, no LLZO was formed but only La_2_Zr_2_O_7_. LLZO only appeared at 650 °C and the intensity of LLZO increased with the deposition temperature. Complete garnet phase formed at 700 °C. Depth profile of the chemical composition measured by time‐of‐flight‐secondary ion mass spectrometry (ToF‐SIMS) revealed a side‐effect, e.g. interfacial diffusion. A clear interface between deposited LLZO and the substrate can clearly be seen (deposited at 150 °C in Figure 8d), while a diffusion interface was observed when the electrolyte film was deposited at 700 °C. Very severe diffusion of Li, Al, and La to substrate and CsFe to the film side were detected when deposited at 800 °C.

**Figure 9:**
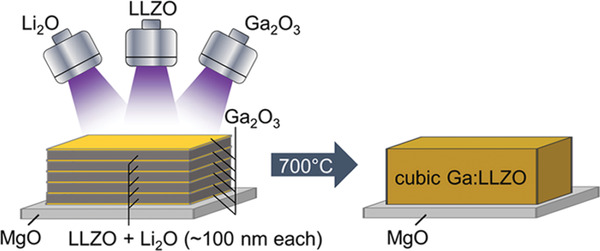
Sputtering of LLZO using multi‐sputtering target. Reproduced with permissioin.^[^ ^111^ ^]^ Copyright 2018, AmericanChemical Society.

Li loss during sputtering is a common phenomenon. To compensate the Li loss, excess amount of Li should be added in the target sintering processing. As a consequence, the target with nonstoichiometry is produced. Another more flexible method is to use rf‐co‐sputtering where multitargets are used. One of the examples is shown in **Figure** **9** in which an additional target of Li_2_O is used together with the LLZO target. The advantage of using the multitarget is easy to control of Li content by simply adjusting the rf‐sputtering power of Li_2_O target. This idea can also be extended to doping of LLZO with more targets such as Ga doping using Ga_2_O_3_ individual target instead of preincorporation of a fixed amount of Ga or other elements in LLZO. In this particular example, the dopant was deposited as a separate layer and incorporation of doping elements was achieved by subsequent postannealing process.

#### 3.2.2 NASICON Type of Electrolyte

**Figure 10:**
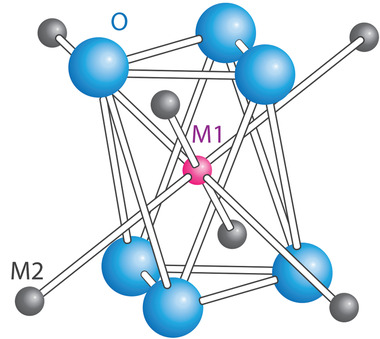
Oxygen environmental of M1 Li showing bottleneck of the triangle between M1 and M2 Li^+^ where the blue spheres represent oxygen atoms, Li^+^ at M1 and M2 sites are presented by the pink sphere and grey spheres, respectively. Reproduced with permission.^[^ ^116^ ^]^ Copyright 1998, American Chemical Society.

The name of NASICON is originated from words “Na superionic conductor” back to about 1976.^[^ ^112^, ^113^ ^]^ A typical example of the “real” NASICON is Na_3_Zr_2_Si_2_PO_12_. Later, the same type of structure was also discovered in Li ion‐based electrolytes, and therefore the name of NASICON is no longer limited to Na superionic conductor but is meant a type of crystal structure. The general formula of NASICON‐type of Li ion electrolyte has the general formula of LiM_2_(PO_4_)_3_ where the tetravalent M = Ti^4+^,^[^ ^114^, ^115^, ^116^, ^117^, ^118^ ^]^ Ge^4+^,^[^ ^116^, ^117^ ^]^ Sn^4+^,^[^ ^116^, ^119^ ^]^ Hf^4+^,^[^ ^116^, ^120^ ^]^ Zr^4+^,^[^ ^115^, ^117^, ^121^ ^]^ among which Ti^4+^, Ge^4+^, and Zr^4+^ has been received great attention due to their high ionic conductivity and easy synthesis. The NASICON framework with a rhombohedral $R\bar{3}c$ symmetry is built up of MO_6_ octahedra and PO_4_ tetrahedra with corner shared.^[^ ^122^ ^]^ Li ions reside in two sites, Li(1) (6b) that is in an elongated octahedral oxygen environment at the intersection of three migration channels, and Li(2) (36f) that is in an 8–10 oxygen environment at each bend of the migration channels.^[^ ^116^ ^]^ When a Li^+^ migrates from Li(1) to Li(2) site, it must pass through the bottleneck of the triangles shown in **Figure** **10**.

**Figure 11:**
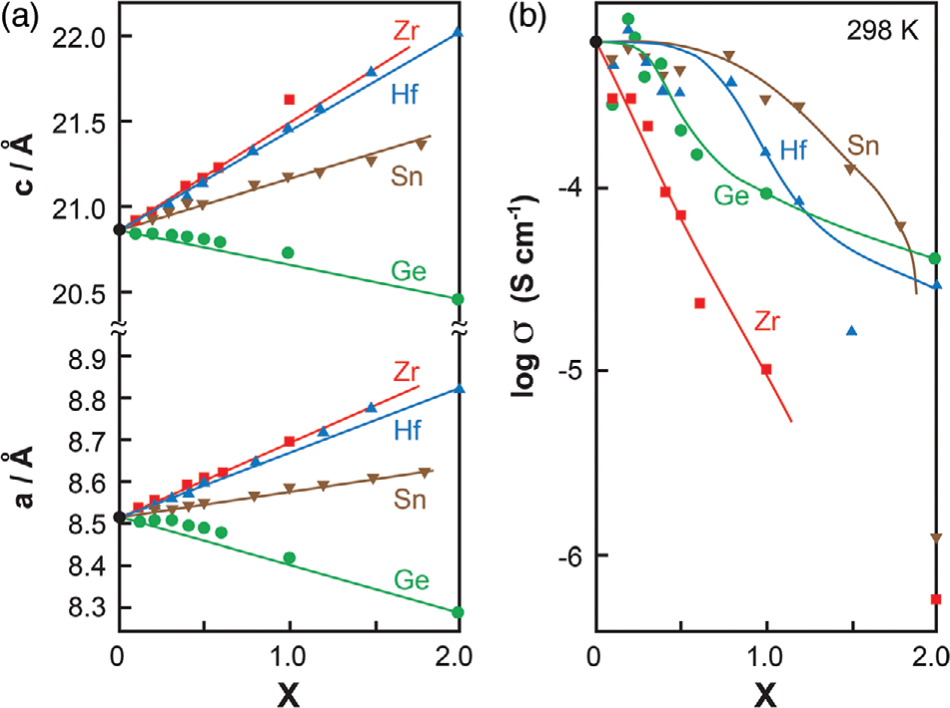
Variation of lattice parameters and ionic conductivity of LLTO at different amount of dopants: a) changes of lattice parameter with ion radii and b) ionic conductivity of LiTi_2‐_ *_x_* M*_x_* (PO_4_)_3_. Reproduced with permission.^[^ ^115^ ^]^ Copyright 1993, IOP Publishing.

The lattice parameters change with different ion sizes at MO_6_ octahedra. The ion radii of Ge^4+^, Ti^4+^, Sn^4+^, Hf^4+^, and Zr^4+^ are 53, 61, 69, 71, and 72 pm, respectively. Taking Ti^4+^ as a reference, the substitution of Ti^4+^ with different amount of Ge^4+^, Sn^4+^, Hf^4+^ and Zr^4+^ in LiTi_2‐_ *_x_* M*_x_* (PO_4_)_3_ clearly shows change of the lattice parameters are associated with the radii as shown in **Figure** **11**a.^[^ ^115^ ^]^ Among the five different tetravalent ions, LiTi_2_(PO_4_)_3_ (LTP) demonstrates the highest ionic conductivity. However, there is no direct correlation between ionic conductivity and the lattice parameters. For example, Zr^4+^ radius is about 15% larger than that of Ti^4+^, but the ionic conductivity of LiZr_2_(PO_4_)_3_ (LZP) is about three order of magnitude lower than (Figure 11b).

**Figure 12:**
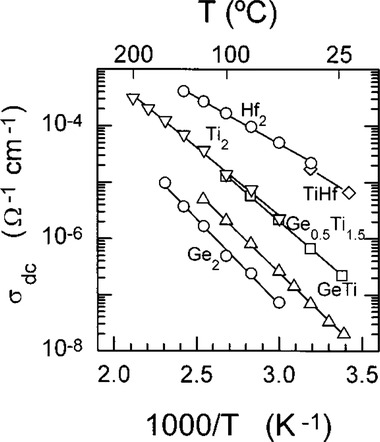
Conductivities measured by dc method of LiM_2_(PO_4_)_3_ where ○ Ge_2_ = LiGe_2_(PO_4_)_3_, ∆ GeTi = LiGeTi(PO_4_)_3_, □ Ge_0.5_ = LiGe_0.5_Ti_1.5_(PO_4_)_3_, ∇ Ti_2_ = LiTi_2_(PO_4_)_3_, ◇TiHf = LiTiHf(PO_4_)_3_, and ○ Hf_2_ = LiHf_2_(PO_4_)_3_. Reproduced with permission.^[^ ^116^ ^]^ Copyright 1998, American Chemical Society.

Since there is only one Li^+^ per formula, the ionic conductivity of LiM_2_(PO_4_)_3_ is about 10^−7^–10^−5^ S cm^−1^ depending on kind of tetravalent ions used. **Figure** **12** summarizes changes of ionic conductivity measured by dc polarization method when tetravalent elements were used to dop in MO_6_ octahedra, among which Ge^4+^ shows the least contribution to Li^+^ conductivity whereas Hf demonstrates the best effect which is somewhat different from those obtained from impedance method (Figure 11b).^[^ ^116^ ^]^ It should be noted that the conductivity measured is a total conductivity including bulk, grain boundary, and interface contributions since it was measured by a dc polarization method which is unable to rule out different contributions.

It is well known that the ionic conductivity is dependent on the mobility of the mobile ions while the mobility is associated with concentration of Li^+^ as well as vacancy sites of Li ions. To increase Li^+^ concentration, the tetravalent M^4+^ can be considered to be a substitute by lower valency ions such as trivalent and / or bivalent ions. The defect reaction of this kind of doping can be therefore written as:

(9) $${S^{3+}}_{M}^{*}={S^{3+}}_{M^{4+}}^{\prime }+{\mathrm{Li}}_{\;i}^{•}$$

and

(10) $${S^{2+}}_{M}^{*}={S^{2+}}_{M^{4+}}^{\prime \prime }\;+2{\mathrm{Li}}_{\;i}^{•},$$

leading to Li_1+_ *_x_* S*_x_* M_2‐_ *_x_* (PO_4_)_3_and Li_1+2_ *_x_* S_x_M_2‐_ *_x_* (PO_4_)_3_ for trivalent and bivalent substitution, respectively.

**Figure 13:**
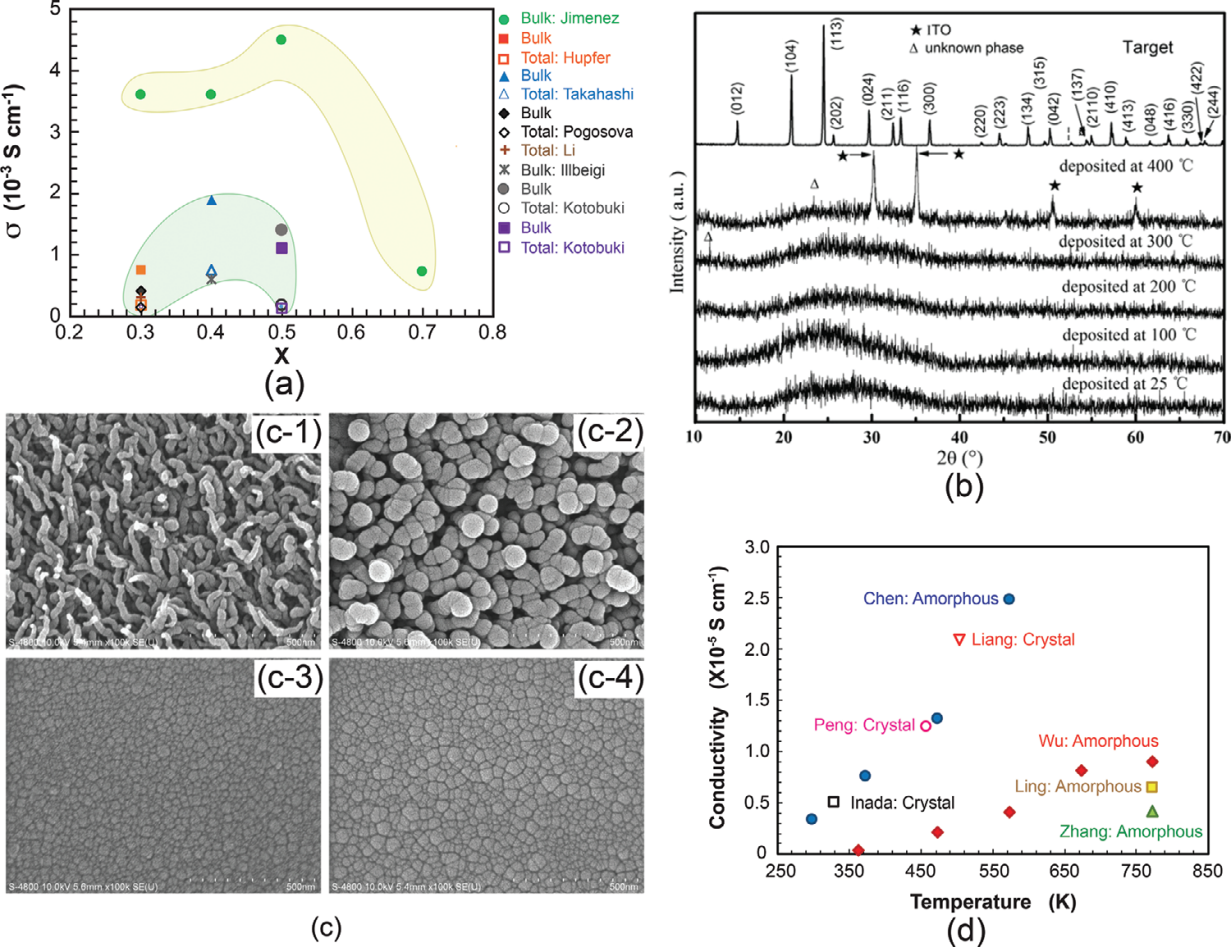
a) Influence of Li concentration of Li_1+_ *_x_* Al*_x_* Ti_2‐_ *_x_* (PO_4_)_3_ showing the trend of increase in ionic conductivity with increase in Li concentration (Jimenez,^[^ ^135^ ^]^ Hupfer,^[^ ^136^ ^]^ Takahashi,^[^ ^137^ ^]^ Pogosova,^[^ ^138^ ^]^ Li,^[^ ^74^ ^]^ Illbeigi,^[^ ^139^ ^]^ Kotobuki^[^ ^140^ ^]^), b) structural evolution of Li_1.3_Al_0.3_Ti_1.7_(PO_4_)_3_ films which were deposited using an rf‐PVD at different substrate temperatures, c) morphologies of the LATP thin films deposited at different temperatures: c‐1) 25 °C, c‐2) 100 °C, c‐3) 200 °C and c‐4) 300 °C. b,c) Reproduced withpermission.^[^ ^141^ ^]^ Copyright2011, Elsevier. d) Ionic conductivities of NASICON‐structured amorphous thin film electrolytes deposited at different temperatures (Chen (Li_1.3_Al_0.3_Ti_1.7_(PO_4_)_3_; rf‐sputtering),^[^ ^141^ ^]^ Wu (Li‐Ti‐Si‐P‐O‐N; rf‐sputtering),^[^ ^142^ ^]^ Ling (Li_1.3_Al_0.3_Ti_1.7_(PO_4_)_3_; rf‐sputtering),^[^ ^143^ ^]^ Zhang (Li_1.4_Si_0.4_Ti_1.6_(PO_4_)_3_; rf‐sputtering),^[^ ^144^ ^]^ Peng (Li_1.3_Al_0.3_Ti_1.7_(PO_4_)_3_; hydrothermal),^[^ ^145^ ^]^ Liang (Li_1‐_ *_x_* Ti_2‐_ *_x_* Ga*_x_* (PO_4_)_3_; hydrothermal),^[^ ^146^ ^]^ Inada (Li_1.5_Al_0.5_GE_1.5_(PO_4_)_3_; aerosol)^[^ ^324^ ^]^).

Many trivalent ions such as Al^3+^,^[^ ^123^, ^124^, ^125^, ^126^, ^127^ ^]^ Fe^3+^,^[^ ^128^, ^129^ ^]^ Cr^3+^,^[^ ^128^, ^130^ ^]^ La^3+^,^[^ ^126^, ^131^ ^]^ B^3+^,^[^ ^126^ ^]^ Ga^3+^,^[^ ^132^ ^]^ Sc^3+^,^[^ ^132^ ^]^ and Y^3+^,^[^ ^133^ ^]^ and bivalent ions such as Ca^2+^,^[^ ^121^ ^]^ Sr^2+^,^[^ ^134^ ^]^ etc are used. One of often used substitutions is Al^3+^. The typical compositions include Li_1+_ *_x_* Al*_x_* Ge_2‐_ *_x_* (PO_4_)_3_ and Li_1+_ *_x_* Al*_x_* Ti_2‐_ *_x_* (PO_4_)_3_, and Li_1+_ *_x_* Al*_x_* Zr_2‐_ *_x_* (PO_4_)_3_. Substitution of the tetravalent ion by the trivalent Al leads to increase in Li concentration and hence ionic conductivity and the ionic conductivity is peaked at about *X* = 0.4–0.5 as shown in **Figure** **13**a.

Based on various compositions of NASICON, the thin film electrolytes with crystalline and amorphous have been investigated. The NASICON electrolyte in an amorphous state can be easily fabricated by rf‐sputtering, PLD, and CVD at different processing conditions. However, different deposition parameters may cause the differences in crystallinity, morphology, and hence conductivity. Taking Li_1.3_Al_0.3_Ti_1.7_(PO_4_)_3_ as an example, the films deposited at the substrate temperature below 400 °C all show amorphous structures judged from XRD measurement as depleted from Figure 13b.^[^ ^141^ ^]^ The one deposited at 400 °C reveals some crystalline peaks while they are unable to be identified to be from LATP but from the bottom electrode, demonstrating that a crystalline LATP would only be obtained at the substrate temperature above 400 °C. The EIS measurement of the film deposited at 400 °C showed two semicircles, probably implying the formation of crystalline interfaces. It should be carefully noted that although all electrolytes prepared below 400 °C possess the amorphous structure, morphologically they are entirely different. Deposition at room temperatures resulted in nanowire‐like 1D growth (Figure 13c‐1), which was associated with deposition of seeds at the very beginning of deposition.^[^ ^141^ ^]^ This phenomenon is like the growth of crystalline where the normal surfaces of the seeds have high surface energy. To minimize the energy increase, the new atoms will land on the surfaces with the highest surface energy. At the relatively high temperature of 100 °C, the film not only grew normally but also literally (Figure 13c‐2). Continuous expansion literally led to a flat surface when the substrate reached 200 °C and above (Figure 13c‐3,4). It should mention that a glance of the surface morphologies of the film deposited at 200 °C and above looks like crystalline since there are “grains” and “grain boundaries.” However, it is not. The grain‐like structure is caused by the merging of amorphous clusters where the middle of the clusters is higher than their boundaries. The “grain boundaries” are just the valleys of the amorphous clusters.

The ionic conductivity which is about two orders of magnetite lower than that of its bulk counterpart is deposition temperature dependent, namely crystallinity dependent. Figure 13d reveals ionic conductivities of amorphous LATP and LSTP deposited at various temperatures. A conclusion that can be drawn based on this observation is that the higher the deposition temperature, the higher the conductivity will be. In general, the ionic conductivity of the amorphous thin films is about 10^−6^–10^−5^ S cm^−1^ even it was deposited at 773 K. In order to achieve a high ionic conductivity similar to the bulk type of electrolyte, high temperature postannealing is necessary, but it will cause an interfacial reaction.

#### 3.2.3 Perovskite Type of Electrolyte

Perovskite type of electrolyte is another Li ion conductor that is widely investigated. The perovskite structure has a general formula of ABO_3_ where A site is occupied by bivalent ions that is surrounded by 12 oxygen atoms and B site by tetravalent ones surrounded by 6 oxygen atoms. The advantage of the perovskite structure is its ion radii tolerance at A‐ and B‐sites and hence the bivalent and tetravalent ions can be substituted by different valent ions.^[^ ^148^, ^149^ ^]^

**Figure 14:**
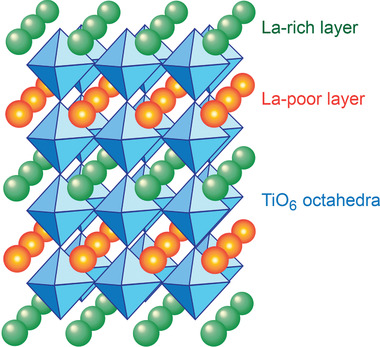
Perovskite structure of Ln*_x_* Li*_y_* TiO_3_ showing alternate Ln layers where Ln = La, Pr, Nd, Sm.

**Figure** **14** shows the perovskite structure where both Ln and Li shear the same A‐sites but with different concentration in their alternate layers.^[^ ^148^ ^]^ When A‐sites are occupied by trivalent ions, vacancies at A‐sites will form according to

(11) $${M^{3+}}_{M}^{*}={M^{3+}}_{A}^{•}\;+\frac{1}{2}V_{A}^{\prime \prime }.$$

The density of A‐site vacancies, or cation deficiency at the A‐sites can be adjusted by inserting different amount of Li^+^ according to

(12) $$a{M^{3+}}_{M}^{*}+b\;L{i^{+}}_{\mathrm{Li}}^{*}=\;a{M^{3+}}_{A}^{•}+bL{i^{+}}_{A}^{\prime }+\frac{\left(a-b\right)}{2}V_{A}^{\prime \prime }$$

**Figure 15:**
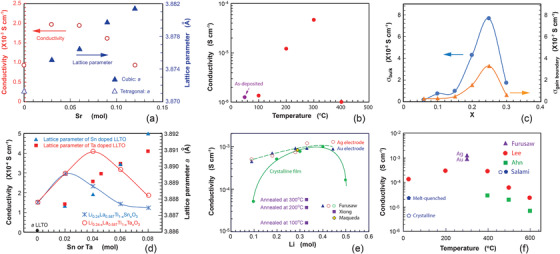
Influence of dopants on the conductivity: a) changes of lattice parameter as well as ionic conductivity of bulk Li_0.33+_ *_x_* La_0.56−_ *_x_* Sr*_x_* TiO_3_ after A‐site substitution of Sr (data from^[159^ ^]^), b) changes of ionic conductivity of rf‐sputtered Li_0.33+_ *_x_* La_0.56−_ *_x_* Sr*_x_* TiO_3_ (data from^[161^ ^]^), c) changes of bulk and grain boundary ionic conductivity at different concentration of Al^3+^, (data from^[168^ ^]^), d) influence of B‐site substitution on the lattice parameters and ionic conductivities of Li_0.24_La_0.587_Ti_1‐_ *_x_* Sn*_x_* O_3_ and Li_0.24‐_ *_x_* La_0.587_Ti_1‐_ *_x_* Ta*_x_* O_3_ (data from^[^ ^162^ ^]^), e) conductivity of La_(2/3) − *x*_Li_3*x*_⊡_(1/3) − 2*x*_TiO_3_ at different Li^+^ concentration and processing temperatures (data from^166^, ^167^, ^168^ ^]^), and f) conductivity of Li_0.5_La_0.5_TiO_4_ thin‐film electrolytes deposited by PLD at various temperatures (data from^166^, ^171^ ^]^ and^[^ ^172^ ^]^ where PLD was used, and ^[176^ ^]^ where melt‐quench was used.)

Therefore, the composition of the perovskite‐type of electrolyte with space group *P*4/*mmm* can be written as

(13) $$Ln_{\left(2/3\right)-x}Li_{3x}{⊡}_{\left(1/3\right)-2x}\mathrm{Ti}O_{3}$$

where Ln = La, Pr, Nd, Sm, and ⊡ represents vacancies at A sites.^[^ ^148^, ^150^ ^]^ Ti atoms occupy B‐sites forming TiO_6_ octahedra. Li^+^ migrates through four adjacent BO_6_ octahedra bottleneck (four oxygen atoms) to an adjacent vacancy.^[^ ^151^, ^152^ ^]^ The bottleneck dimensions differ from layer to layer due to slightly BO_6_ tilting. It has been noted that the use of smaller radii of ions in Ln sites will lead to a structural transformation from a cubic lattice to an orthorhombic one, resulting in a decreased ionic conductivity.^[^ ^151^ ^]^ Through using the nudged elastic band (NEB), the highest Li ionic conductivity can be obtained when *x* = 0.067,^[^ ^153^ ^]^ while experimental studies revealed that the maximum ionic conductivity can be obtained between *x* = 0.10 and 0.13.^[^ ^154^, ^155^ ^]^ The best ionic conductivity of the bulk electrolyte at the room temperature is about 10^−4^–10^−3^ S cm^−1^. The bivalent Sr^2+^ has also been used to dope at A site.^[^ ^156^, ^157^ ^]^ Substitution of La with low valency ions such as Sr^2+^, Ba^2+^, and Ca^2+^ can cause the change in the Li ion concentration and at the same time a structural change from a tetragonal to a cubic structure. **Figure** **15**a shows changes of lattice parameters and ionic conductivity of Li_0.33+_ *_x_* La_0.56−_ *_x_* Sr*_x_* TiO_3_ with different amount of Sr^2+^.^[^ ^156^ ^]^ With substitution as little as 0.05 mol, incorporation of Sr^2+^ caused structural transformation from its tetragonal to a cubic structure. The lattice parameter of the cubic Li_0.36_La_0.53_Sr_0.03_TiO_3_ is larger than lattice parameter, *a*, of the tetragonal structure, and further increases with the amount of Sr^2+^. While the ionic conductivity increases at a small dopant level and decreases due to reduction of vacancies although the lattice parameter increases monoclinically.

Li_0.43_La_0.457_Sr_0.1_TiO_3_ thin film fabricated by rf‐sputtering followed by postannealing at different temperatures also showed a strong positive effect from Sr^2+^ dopant. The ionic conductivity of the as‐deposited Li_0.43_La_0.457_Sr_0.1_TiO_3_ thin film at 50 °C was only 1.263 × 10^−6^ S cm^−1^ but increased to with the postannealing temperature and reached a maximum value of 4.631 × 10^−5^ S cm^−1^ after annealing at 300 °C although it still displayed an amorphous structure from XRD characterization, and the conductivity sharply dropped at a higher annealing temperature of 400 °C (Figure 15b).^[^ ^158^ ^]^

To improve stability and conductivity, B‐site substitution has also been widely practiced, including use of trivalent ions Al^3+^,^[^ ^159^ ^]^ Co^3+^,^[^ ^159^ ^]^ In^3+^,^[^ ^159^ ^]^ tetravalent ions Zr^4+^,^[^ ^160^, ^161^ ^]^ Sn^4+^,^[^ ^162^ ^]^ and pentavalent ions Ta^4+^,^[^ ^160^, ^161^, ^162^ ^]^ V^5+^,^[^ ^163^ ^]^ and Nb^5+^.^[^ ^163^, ^164^ ^]^ Partial substitution of Ti^4+^ by trivalent ions such as Al^3+^ causes increased charge carriers, hence resulting in an enhanced ionic conductivity. Because of the increase in the concentration of Li^+^, the concentration of vacancies reduced. When the concentration of vacancy sites is below a certain critical value, the ionic conductivity is noted to decrease.^[^ ^165^ ^]^ Figure 15c shows the changes of bulk ionic and grain boundary conductivity of La_2/3_Li*_x_* Ti_1‐_ *_x_* Al*_x_* O_3_ at different amounts of Al^3+^, which clearly demonstrates the influence of Li^+^ and Li vacancies concentration on the ionic conductivities.^[^ ^165^ ^]^

The substitution of Ti^4+^ by tetravalent ions will cause changes neither in Li^+^ nor vacancy concentration, namely

(14) $${M^{4+}}_{M}^{*}={M^{4+}}_{\mathrm{Ti}}^{*}\;$$

but will induce the change of the bottleneck size for Li ions migration. If the pentavalent ion is used, it will result in the increase in vacancies via

(15) $${M^{5+}}_{M}^{*}={M^{5+}}_{\mathrm{Ti}}^{•}\;+V_{\mathrm{Li}}^{\prime }$$

Substitution of Ti^4+^ respectively by Sn^4+^ and Ta^5+^ displays an increase in the ionic conductivity as displayed in Figure 15d.^[^ ^162^ ^]^ The increase in the conductivity of LLTO doped by Sn can be attributed to increase in bottleneck while at a high dopant level there was a decrease in the conductivity even though the lattice parameter continuously increased. It seems that the vacancy level of Li_0.24_La_0.587_TiO_3_ is not optimized, ionic conductivity can be further increased with the increase in Li vacancies with substitution of Ti^4+^ by Ta^5+^.

Figure 15e reveals the variation of ionic conductivity at different Li concentration of pristine L La_(2/3) − *x*_Li_3*x*_⊡_(1/3)−2_ *_x_*. TiO_3_. Three phenomena can clearly be observed: a) the ionic conductivity increases with Li^+^ concentration and is peaked at about 0.4, b) the higher the postannealing temperature, the higher the ionic conductivity will be, and c) the amorphous structured LLTO has higher ionic conductivity in comparison with their crystallines.^[^ ^166^, ^167^, ^168^ ^]^ The LLTO thin‐film electrolyte prepared by rf‐sputtering at room temperature has a very low ionic conductivity of 0.71 × 10^−5^ (not shown in Figure 15e). Annealing at different temperature led to increase in the conductivity. Whereas the amorphous‐structured thin films prepared by PLD by Furusawa et al.^[^ ^166^ ^]^ showed very high conductivity. However, after high‐temperature crystallization at 1350 °C, the thin film electrolytes in a crystal form surprisingly had lower ionic conductivity compared with their amorphous counterparts. TEM study revealed that the nanocrystals were embedded in the amorphous structure even though XRD showed an amorphous state, which could be a reason why PLD deposited LLTO had higher an ionic conductivity since it is possible to have tiny clusters to land together with atoms on the substrate. The interfaces between the amorphous and the nanocrystal domains may act as mobile careers’ sites enabling the hoping of Li ions.

Like garnet‐ and NASICON‐structured thin films, deposition of LLTO thin‐film electrolyte at high temperature is essential in order to achieve a crystal structure. Although the temperature of deposition is composition and operation parameters (such as oxygen partial pressure, rf‐sputtering power density etc) dependent, the minimum temperature for pristine LLTO must be about 700 °C.^[^ ^169^ ^]^ Well‐crystallized perovskite Li_0.5_La_0.5_TiO_4_ could only be obtained by further post‐annealing at 1350 °C.^[^ ^166^ ^]^ Deposition using PLD shows that the crystalline phase had yet been achieved at a deposition temperature of 700 °C.^[^ ^168^, ^170^ ^] ^The systematic investigations of Li_0.5_La_0.5_TiO_4_ films that were deposited at various temperatures have demonstrated that the high ionic conductivity comparable to bulk ceramic one can be achieved in an amorphous state. Wide deposition temperature ranges were studied by Lee^[^ ^171^ ^]^ and Ahn,^[^ ^172^ ^]^ showing that with proper control of deposition, the ionic conductivity of the thin film electrolyte deposited at room temperature was about 10^−4^ S cm^−1^ and increased with deposition temperature and was maximized at the deposition temperature between 300 and 400 °C as shown in Figure 15f,^[^ ^171^ ^]^ while data from Ahn et al. showed a continuous decrease, which is consistent with Lee's finding. The highest ionic conductivity is from Furusaw's work in which the LLTO thin film was prepared by PLD at room temperature followed by a postannealing at 300 °C. The higher conductivity is probably attributed to compositional change since the composition is off stoichiometry of Li_0.5_La_0.5_TiO_4_, resulting in formation of Li vacancies and hence a high conductivity.^[^ ^166^ ^]^ Two kinds of blocking electrode were used, one of which is Ag and another Au in this study. As a comparison, the ionic conductivity of bulk glass‐ceramic containing about 60% crystalline is included in Figure 15f from Salami et al. The bulk glass‐ceramic electrolyte was fabricated by melt‐quench method using a rolling machine and its ionic conductivity is 1.3 × 10^−5^ S cm^−1^, higher than 4.733 × 10^−6^ S cm^−1^ of the crystalline counterpart.^[^ ^173^ ^]^

## 4 Thin Film Electrodes for ATFBs

As the Li ion reservoirs, namely cathode and anode, the thin film electrodes have been intensively explored. There are vast choices for the positive electrode while there are much less possible selections for the negative electrode although many of materials for anode thin electrode were studied. There are a number of challenges in order to really realize success of ATFBs: a) how to successfully achieve the charge transfer between the electrolytes and the electrodes, and the migration of ions from one Li ion reservoir to another since there is no electron‐conducting materials in the electrodes such as carbon and also no ion‐conducting electrolyte, b) how to achieve low substrate temperature deposition but still possessing a good crystallinity, and c) how to minimize interfacial strains between the electrode and the electrolyte.

**Table** **1** tabulates different types of electrode materials that have been studied as the anodes and cathodes in the ATFBs as well as in the half‐cells. The anode studied can be categorized into three types: intercalation, conversion, and alloying, whereas typical cathode materials are all intercalation type.

**Table 1:** Electrode materials for anode

No	Name	Composition of electrode^a)^	Potential (V vs Li^+^/Li)	Specific capacity [µAh cm^−2^µm^−1^]	Deposition method/temperature [°C]	Refs.
1	Lithium metal	Li	0	3686 mAh g^−1^	Vacuum evaporation	^[^ ^174^, ^175^ ^]^
2	Miultiwall carbon nanotubes	MWCNT (LE)	0.7–0	78 mAh g^−1^	Chemical vapor deposition	^[^ ^176^ ^]^
3	Lithium nickel vanadate	LiNiVO_4_ (LE)	1.8–0	400	rf‐sputtering/*T*_r_	^[^ ^177^ ^]^
4	Nickel vanadate	Ni_2_V_2_O_7_ (LE)	0.8	750 mAh g^−1^	Electron beam/ postannealed 863 K	^[^ ^178^ ^]^
5	Silicon	Si	0.3	3500 mAh g^−1^	rf‐sputtering/*T*_r_	^[^ ^179^ ^]^
5	Silicon and germanium alloy	Si_1‐_ *_x_* Ge*_x_* (LE)	0.75–0	1217–2121 mAh g^−1^	Glancing angle deposition/*T*_r_	^[^ ^183^ ^]^
6	Silicon and copper alloy	Si–Cu (LE)	0.3	1100–1400 mAh g^−1^	dc‐sputtering and rf‐sputtering/*T*_r_, postannealed 200	^[^ ^184^, ^185^, ^186^ ^]^
7	Si–Ti–Fe alloy	Si–Ti–Fe (LE)		1400 mAh g^−1^	rf‐sputtering/*T*_r_	^[^ ^187^ ^]^
8	Germanium–titanium nitrate alloy	Ge–TiN (LE)	1.5–0	1180–1550 mAh g^−1^	Dual sputtering/*T*_r_	^[^ ^188^ ^]^
9	Sn–Cu alloy	Sn–Cu (LE)	0.4	940 mAh g^−1^	Electrodeposition/ *T*_r_, postannealed 200	^[^ ^184^ ^]^
10	Sn–Co–Ni alloy	Sn–Co–Ni (LE)	0.4	717 mAh g^−1^	Electroplating/*T*_r_	^[^ ^189^ ^]^
11	Cu–Sb alloy	Cu_2_Sb (LE)	0.8	940 mAh g^−1^	Electrodeposition/*T*_r_	^[^ ^190^ ^]^
12		FeF_2_ (LE)	1.5	49 mAh g^−1^	PLD/*T*_r_	^[^ ^191^ ^]^
13	Tin oxide	SnO_2_(LE)	0.5–0	600 mAh g^−1^	Photochemical vapor deposition/ 473–573 K	^[^ ^192^ ^]^
13		SnO_2_–Se (LE)	1.5–0	958 mAh g^−1^	PLD/*T*_r_	^[^ ^193^ ^]^
14	Bismuth iron oxide	BiFeO_3_ (LE)	1.5–0	770 mAh g^−1^	PLD/600	^[^ ^194^ ^]^
15	Iron oxide	Fe_2_O_3_ (LE)	0.9	905 mAh g^−1^	PLD/*T*_r_	^[^ ^195^ ^]^
15	Iron phosphides	FeP*_y_* (LE)	2–0	275 mAh g^−1^	Electrodeposition/*T*_r_	^[^ ^196^ ^]^
24	Gallium selenide	Ga_2_Se_3_ (LE)	1.25	700 mAh g^−1^	Coevaporation/400	^[^ ^197^ ^]^
16	Tantalum oxide	Ta_2_O_5_	0.8	100 mAh g^−1^	PLD/*T*_r_	^[^ ^198^ ^]^
18	Lithium titanate	Li_4_Ti_5_O_12_ (LE)	1.55	60	Sol‐gel, annealed 600–800	^[^ ^199^ ^]^
19				170 mAh g^−1^	Dip‐coating, annealed 650	^[^ ^200^ ^]^
20				97	Magnetron‐sputtering/500–700	^[^ ^201^ ^]^
21				152 mAh g^−1^	PLD/*T*_r_, postannealed 700	^[^ ^202^ ^]^
22				25	dc‐ion beam sputtering/600–700	^[^ ^203^ ^]^
23		Li_4/3_Ti_5/3_O_4_(LE)	1.55	40	Sol‐gel, annealed 600	^[^ ^204^ ^]^
25	Titanium dioxide	TiO_2_	1.8	148 mAh g^−1^	Polyvinylpyrrolidone sol‐gel/annealed 600	^[^ ^205^ ^]^
26	Titanium oxysulfide	TiOS	2.25		dc‐reactive sputtering/*T*_r_	^[^ ^206^ ^]^
27	Titanium oxysulfide	TiSi*_x_* O*_y_*			rf‐sputtering/*T*_r_	^[^ ^174^ ^]^
28	Titanium sulfide	TiSi_2_	2.5		rf‐sputtering/*T*_r_	^[^ ^175^ ^]^
29						^[^ ^207^ ^]^
30	Silver iodide	AgI	2.1			^[^ ^3^ ^]^

^a)^ ATFBs were tested based on full cells unless otherwise stated as “LE” indicating half‐cell using the liquid electrolyte.

### 4.1 Anode Thin Film Electrodes

Table 1 tabulates various electrode materials for anode, among which from potential point of view, the ideal anode would be Li metal since it has a potential ‐3.04 versus H^+^ with a theoretical capacity 3861 mAh g^‐1^), leading to the highest achievable voltage. Since the melting temperature of Li is as low as 180.5 °C, one of the most suitable deposition methods is thermal evaporation.^[^ ^180^ ^]^ The thickness can be in the range of sub‐micrometer since the revivor of Li ions is from the cathode. Deposition of Li metal is always a challenge due to poor wettability of Li with almost all ceramics.^[^ ^181^ ^]^ To increase wettability, Wu et al. innovatively introduced carbon particles into molten Li, resulting in a large decrease in interfacial energy between the molten Li and coated counterpart. This facial method has been very successfully used in garnet‐type solid electrolyte and Cu foil.^[^ ^182^ ^]^

The typical examples of the alloying type of anode are Si–Ge and Si–Cu alloys,^[^ ^183^, ^184^, ^185^, ^186^ ^]^ and the typical intercalation types of anodes are TiO_2_ and Li_4_Ti_5_O_12_.^[^ ^199^, ^200^, ^201^, ^202^, ^203^, ^204^, ^205^ ^]^ Both alloy and conversion types of anode possess the highest capacity but with the largest volumetric change during galvanotactic cycles. Among all alloying type of electrode materials, Si demonstrates the highest specific capacity through the alloying process

(16) $$\mathrm{Si}+xLi^{+}+xe^{-}=Li_{x}\;\mathrm{Si}$$

**Figure 16:**
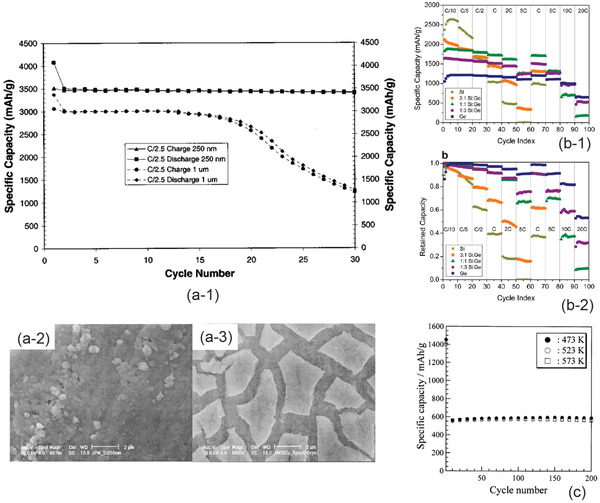
a) Electrochemical performance and morphologies of Si thin film: a‐1) galvanostatic cyclability of the Si thin films with thickness 250 nm and 1 µm, a‐2) morphology as deposited, and a‐3) the morphology after 30 galvanostatic cycles. Reproduced with permission.^[^ ^179^ ^]^ Copyright 2003, IOP Publishing. b) Electrochemical performance of Si_1‐_ *_x_* Ge*_x_*: b‐1) specific capacity and b‐2) capacity retention at different Ge concentrations. Reproduced with permission.^[^ ^183^ ^]^ Copyright 2013, American Chemical Society. c) Cyclability of SnO_2_ thin film deposited by photochemical vapor deposition at a current density of 50 μA cm^−2^. Reproduced with permission.^[^ ^192^ ^]^ Copyright 2001, Elsevier.

A maximum specific capacity of about 4200 mAh g^−1^ can be achieved corresponding to the formation of Li_4.4_Si that leads to an extraordinary volume expansion of about 420%.^[^ ^264^ ^]^ This large volume change will obviously cause pulverization of the Si due to inhomogeneity of intercalation of Li. To minimize compositional difference during charge and discharge, nanothin film electrode was proposed. The cyclabilities of the two Si thin films with a thickness of 250 nm and 1000 nm respectively are compared in **Figure** **16**a‐1.^[^ ^179^ ^]^

Although the Si thin film of 250 nm shows a good electrochemical cyclability, large cracks were observed just after 30 cycles. The as deposited Si film shows a dens morphology (Figure 16a‐2), while a large number of cracks can be seen from the thin film just after 30 charge/discharge cycles, indicating large internal stress (Figure 16a‐3).

Ge is also an alloying type of anode material with a specific capacity of 1600 mAh g^−1^ but less volume change during the galvanotactic cycle. Therefore, alloy between Si and Ge may prevent the formation of microcracks. Figure 16b shows the specific capacity at different rates and the rate capability of Si_1‐_ *_x_* Ge*_x_* thin film electrode at different amount of Ge prepared by cosputtering using a Si and a Ge targets. Pure Si when *x* = 0 shows the highest specific capacity, but Si–Ge alloys show much better rate capability (Figure 16b‐1). Most importantly the pure Si film surfers from a poor capacity retention due mainly to pulverization (Figure 16b‐2).^[^ ^183^ ^]^

SnO_2_ thin film is an example of conversion type of materials. The conversion is through two steps. In the first step, the SnO_2_ is reduced to Sn by

(17) $$\mathrm{Sn}O_{2}+4Li^{+}+4e^{-}\to \mathrm{Sn}+2Li_{2}O,\;\mathrm{and}$$

Sn will be further alloyed with Li through

(18) $$\mathrm{Sn}+4.4Li^{+}+4.4e^{-}=Li_{4.4}\mathrm{Sn}$$

with a theoretical capacity of 994 mAh g^−1^ and a volume change of 260% if the reduction is not considered.^[^ ^264^ ^]^

Figure 16c shows the cyclability of SnO_2_ thin films that were prepared by photochemical vapor deposition at three different temperatures, namely 473, 523, and 573 K. Except for the film that was deposited at 573 K showing a poor crystallinity, the rest two all showed the amorphous structures. The specific capacity of the first cycle of the SnO_2_ thin film which was tested using an organic electrolyte is about 1450 mAh g^−1^ due to both reduction and alloying processes. Since the reduction is an irreversible, the specific capacity dropped to about 560 mAh g^−1^ starting from the second charge. All three thin‐film anodes display very stable electrochemical performance. Although the difference is small, it is noted that the specific capacities of amorphous structured SnO_2_ films were slightly higher than that deposited at 573 K.^[^ ^192^ ^]^

The advantage of the conversion type of materials over the alloying type of materials is the fact that the formation of irreversible nano Li_2_O grains during the first discharge cycle homogeneously distributes in the anode material, and it will act as buffers that are able to partly accommodate volume change in the further charge and discharge. Although both alloying and conversion types of anode possess the advantage of the high energy storage capacity, large volume, and morphological changes during charge and discharge may cause ATFBs to fade quickly. Therefore, intercalation type of anode thin films is the alternative choice although the specific capacity of the intercalation type of anode materials is much lower.

**Figure 17:**
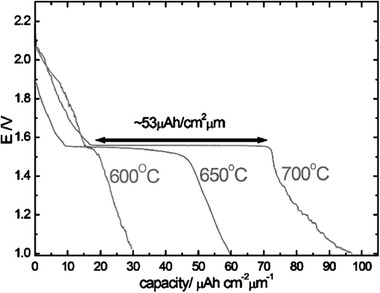
Specific capacity of Li_4_Ti_5_O_12_ thin film electrode that was deposited using magnetron sputtering at three different substrate temperatures. Reproduced with permission.^[^ ^201^ ^]^ Copyright 2005, IOP Publishing.

The spinel‐structured lithium titanate, Li_4_Ti_5_O_12_, is a typical intercalation material with a potential 1.55V versus Li^+^/Li. Three Li ions can be inserted into Li_4_Ti_5_O_12_,

(19) $$Li_{4}Ti_{5}O_{12}+3Li^{+}+3e^{-}=\;Li_{7}Ti_{5}O_{12}$$

having a capacity 175 mAh g^−1^ (60.8 µAh cm^−2^ µm^−1^).^[^ ^201^, ^202^, ^203^ ^]^ Since it is an intercalation material, a good crystallinity is essential to ensure the successful insertion of Li ions. To obtain high crystalline, either the Li_4_Ti_5_O_12_ thin film electrode should be grown at a high deposition temperature or the deposited thin film electrode should be postannealed after thin film growth. Poor crystallinity will result in low specific capacity due to limited Li vacancies. **Figure** **17** reveals the relationship between the discharge profiles and specific capacity at three deposition temperatures. The specific capacity caused by insertion of Li ions (judged from the discharge plateaus) is as low as about 10 µAh cm^−2^ µm^−1^ when the film was deposited at 600 °C and reaches to 53 µAh cm^−2^ µm^−1^ when the film was deposited at 700 °C, which clearly indicates strong correlation between crystallinity and specific capacity.

**Figure 18:**
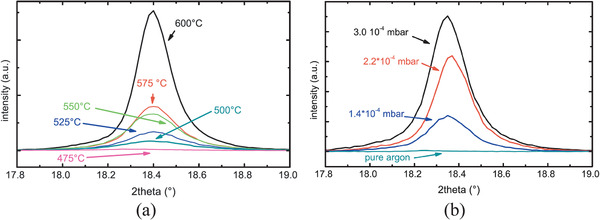
a) Influence of deposition temperature at a constant 3 × 10^−4^ mbar, and b) effect of oxygen partial pressure at a constant temperature of 600 °C on crystallinity of lithium titanate. Reproduced with permission.^[^ ^203^ ^]^ Copyright 2012, Elsevier.

The crystallinity of the films is not only affected by deposition temperature but also strongly affected by the oxygen partial pressure of deposition. **Figure** **18** summarizes changes of crystallinity with the change of deposition temperature (Figure 18a) and with the deposition oxygen partial pressure (Figure 18b). Figure 18a reveals that the crystallinity of the LTO thin film increases with increase in the deposition temperature at a fixed oxygen partial pressure of 3 × 10^−4^ mbar, and the highest crystallinity is achieved when the deposition temperature is as high as at 600 °C. However, even at the same deposition temperature of 600 °C, its crystallinity was largely reduced when oxygen partial pressure was decreased.

Titania, TiO_2_ that has many phases such as anatase, rutile etc. is another intercalation type of anode with a flat discharge potential plateau. Anatase phase demonstrates the best Li^+^ storage capability with a flat potential plateau of 1.7 V versus Li^+^/Li owing to biphasic reaction. Since the anatase TiO_2_ contains one vacancy in its octahedral site per formula, if one Li^+^ can intercalate into the vacancy site through

(20) $$\mathrm{Ti}O_{2}+xLi^{+}+xe^{-}=\;Li_{x}\mathrm{Ti}O_{2}$$

it gives rise to a specific capacity of 336 mA h g^−1^.^[^ ^265^ ^]^ However, due to n‐butyllithium chemical lithiation, a maximum stoichiometric capacity with intercalation of 0.7 Li per formula, Li_0.7_TiO_2_ may only be achieved.^[^ ^266^ ^]^

Rutile TiO_2_ is another consideration but rutile TiO_2_ has much lower electronic conductivity, so the insertion of Li ions is much less leading to a low specific capacity. To increase Li ion storage, nanostructured rutile has been developed.^[^ ^267^ ^]^ Similar to Li_4_Ti_5_O_12_, another characteristics of anatase TiO_2_ is the fact of its low volume change of only about 4% during electrochemical cycles, which will be able to minimize the interfacial strain of the thin film batteries.

### 4.2 Cathode Thin Film Electrodes

There are many candidates for the selection of the cathodes, which mainly include layer‐, spine‐, and olivine‐structured materials as listed in **Table** **2**. Different cathode materials possess different electrochemical performance as well as mechanical responses.

**Table 2:** Electrode materials for cathode

No	Name	Composition of electrode^a)^	Potential (V vs Li^+^/Li)	Specific capacity [µAh cm^−2^µm^−1^]	Deposition method/temperature [°C]	Refs.
1	Titanium oxysulfide	TiOS	2.25		dc‐reactive sputtering/*T*_r_	^[^ ^206^ ^]^
2	Titanium oxysulfide	TiSi*_x_* O*_y_*			rf‐sputtering/*T*_r_	^[^ ^174^ ^]^
3	Titanium sulfide	TiSi_2_	2.5		rf‐sputtering/*T*_r_	^[^ ^175^ ^]^
4						^[^ ^207^ ^]^
5	Silver iodide	AgI	2.1			^[^ ^3^ ^]^
6	Lithium cobalt oxide	LiCoO_2_	3.5–3.9		PLD/*T*_r_, postannealed 400–850	^[^ ^208^ ^]^
7		LiCoO_2_	3.5	50	PLD/*T*_r_, postannealed 800	^[^ ^208^ ^]^
8		LiCoO_2_ LiCo_1‐_ *_x_* Al*_x_* O_2_	3.8		PLD / 300–700	^[^ ^209^ ^]^
9		LiCoO_2_		200 mAh g^−1^	rf‐sputtering/ <180 (amorphous)	^[^ ^210^ ^]^
10		LiCoO_2_ (LE) LiCo_1‐_ *_x_* Al*_x_* O_2_ (LE)		89 mAh g^−1^ 89/3 mAh g^−1^	PLD/700 PLD/600	^[^ ^211^ ^]^
11		LiCoO_2_ (LE)	3.5	195 mC cm^−2^ µm^−1^	PLD/100–300	^[^ ^212^ ^]^
12			3.9		PLD	^[^ ^213^ ^]^
13				52.5	rf‐sputtering (350 °C) with rf plasma treatment	^[^ ^214^ ^]^
14				60	rf‐sputtering/up to 500	^[^ ^215^ ^]^
15				48	Sol‐gel, annealed 600–800	^[^ ^199^ ^]^
16				67	rf‐sputtering/*T*_r_, postannealed 500	^[^ ^216^ ^]^
17		LiNi_0.8_Co_0.2_O_2_	3.7	89 mAh g^−1^	PLD/500–900, postannealed 1000	^[^ ^217^ ^]^
18	Lithium nickel‐manganese‐cobalt oxide	Li(Ni*_x_* Mn*_y_* Co*_z_*)O_2_ (LE)	4.2–3.9	164 mAh g^−1^	rf‐sputtering / postannealed 700	^[^ ^218^ ^]^
19						
20		Li(Ni*_x_* Mn*_y_* Co*_z_*)O_2_	3.8–3.5	33		
21		Li(Ni_1/3_Mn_1/3_Co_1/3_)O_2_		177 mAh g^−1^	PLD/ postannealed 450	^[^ ^219^ ^]^
22	Lithium‐rich nickel‐manganese‐cobalt oxide	*x*Li_2_MnO_3_‐(1‐*x*) Li(Mn_0.375_Ni_0.375_Co_0.25_)O_2_ (LE)	4.5–2		Spin coat/postannealed 450	^[^ ^220^ ^]^
23		Li[Li_0.2_Mn_0.54_Co_0.13_Ni_0.13_]O_2_		45	rf‐sputtering/*T*_r_, post annealed 450–600	^[^ ^221^ ^]^
24		LiCoBO_3_ (LE)	3.5–2.5			^[^ ^222^ ^]^
25		Li_1+_ *_x_* V_3_O_8_	2.9		Li_1+_ *_x_* V_3_O_8_‐polyethylene oxide composite	^[^ ^223^ ^]^
26		Li_1+_ *_x_* V_3_O_8_ (LE)	3.8–1.5	250 mAh g^−1^	rf‐sputtering (mixed amorphous and crystalline)	^[^ ^224^ ^]^
27		LiV_2_O_5_	3.5	30	Plasma enhanced chemical vapor deposition/250	^[^ ^225^ ^]^
28	Lithium manganese oxide	LiMn_2_O_4_ (LE)	4.0		rf‐sputtering/*T*_r_, postannealed 650–800	^[^ ^207^ ^]^
					rf‐sputtering/*T*_r_, postannealed 400	^[^ ^226^ ^]^
					PLD / 500	^[^ ^227^ ^]^
29		LiMn_2_O_4_	4.2	50	Reactive electron beam evaporation/50 (substrate), postannealed 800	^[^ ^228^ ^]^
30				47	rf‐sputtering/*T*_r_, postannealed 750	^[^ ^229^ ^]^
31			4.1–3.9	80 mAh g^−1^	rf‐sputtering/*T*_r_, postannealed 750	^[^ ^230^ ^]^
32		Li*_x_* Mn_2‐_ *_y_* O_4_ (LE)	4.1–3.9	121 mAh g^−1^	rf‐sputtering/*T*_r_ with bias	^[^ ^231^ ^]^
33		LiMn_2_O_4_ (LE)	4.1		rf‐sputtering/*T*_r_, postannealed 700	^[^ ^232^ ^]^
34					Sol‐gel combined with oxygen‐plasma irradiation/ 723, 973 K	^[^ ^233^ ^]^
35					Chemical solution deposition/postannealed 500–750	^[^ ^234^ ^]^
36			4.0, 3.0	1.2 Ah cm^−3^	rf‐sputtering/*T*_r_, postannealed 800	^[^ ^236^ ^]^
37		LiSn_0.0125_Mn_1.975_O_4_ (LE)	4.2	68	rf‐sputtering/*T*_r_, postannealed 500	^[^ ^235^ ^]^
38		LiMn_2_O_4_/SrRuO_3_ (LE)	4.1	125 mAh g^−1^	PLD/650	^[^ ^237^ ^]^
39	Lithium nickel manganese oxide	LiNi_0.5_Mn_1.5_O_4_ (LE)	4.7	155 mAh g^−1^	Electrostatic spray deposition/450, postannealing 700	^[^ ^238^ ^]^
40			4.7	45	Sol‐gel/post annealed 500–700	^[^ ^239^ ^]^
41			4.8	120–155 mAh g^−1^	PLD/600	^[^ ^240^, ^241^, ^242^, ^243^, ^244^, ^245^, ^246^ ^]^
42			5 & 4	120 mAh g^−1^	rf‐sputtering/*T*_r_,	^[^ ^247^ ^]^
43			4.7	50	rf‐sputtering/*T*_r_, postannealed 700 or 750	^[^ ^248^ ^]^
44			4.7	104 mAh g^−1^	rf‐sputtering/*T*_r_, post annealed 600	^[^ ^249^ ^]^
45		LiNi_0.5_Mn_1.5_O_4_	4.8		PLD/ 500, 650	^[^ ^250^ ^]^
46		LiCoMnO_4_ (LE)	5	110 mAh g^−1^	PLD/500	^[^ ^251^ ^]^
47	Lithium iron phosphate	LiFePO_4_ (LE)	3.4		PLD/*T_r_*, postannealed 773 K	^[^ ^252^ ^]^
48				160 mAh g^−1^	PLD/600	^[^ ^253^ ^]^
49					PLD/500	^[^ ^254^ ^]^
50				33	rf‐sputtering/*T*_r_, 500	^[^ ^255^ ^]^
51				56	rf‐sputtering/up to 500	^[^ ^256^ ^]^
52	Lithium cobalt phosphate	LiCoPO_4_	4.8		rf‐sputtering/*T*_r_, postannealed 300–700	^[^ ^257^ ^]^
53	Vanadium pentoxide	V_2_O_5_	3.7		dc sputtering/*T*_r_	^[^ ^207^ ^]^
54			3–2	43	Thermal evaporation	^[^ ^258^ ^]^
55		V_2_O_5_ (LE)	3.4		PLD/*T*_r_, 500	^[^ ^211^ ^]^
56					ALD / postannealed 400	^[^ ^259^ ^]^
57	Mangenues dioxide	MnO_2_	3		rf‐sputtering/*T*_r_, postannealed up to 450	^[^ ^260^ ^]^
58	Molybdenum oxide	MoO_3‐_ *_x_*	3–1.9	85	rf‐sputtering/100	^[^ ^261^ ^]^
59	Molybdenum oxysulfide	MoS*_x_* O*_y_*	2.4–1.3		Electrochemical deposition	^[^ ^262^ ^]^

^a)^ATFBs were tested based on full cells unless otherwise stated as “LE” indicating half‐cell using the liquid electrolyte.

#### 4.2.1 Layer‐Structured of Thin Film Electrode

**Figure 19:**
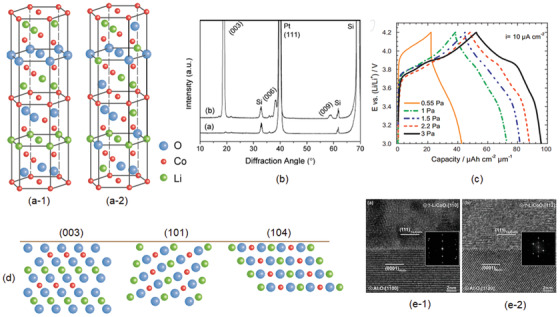
a) Layer‐structured LiCoO_2_ crystal structures: a‐1) high temperature rhombohedral structure and a‐2) low temperature cubic structure. Reproduced with permission.^[^ ^268^ ^]^ Copyright 2012, Elsevier. b) Orientation of thin film prepared by PLD. Reproduced with permission.^[^ ^269^ ^]^ Copyright 2007, Elsevie. c) Influence of oxygen partial pressure used in the deposition on specific capacity. Reproduced with permission.^[^ ^216^ ^]^ Copyright 2012, Elsevier. d) Schematical illustration of three typical orientation, and e) high‐resolution TEM images: e‐1) $\left[1\bar{1}0\right]$ orientated file and e‐2) $\left[11\bar{2}\right]$ orientated film. d,e) Reproduced with permission.^[^ ^270^ ^]^ Copyright 2012, Elsevier.

LiCoO_2_ that has layered rhombohedral structure with a space group $R\bar{3}m$ at high temperature (**Figure** **19**a‐1) and slightly distorted cubic structure with a space group *Fd*3*m* at lower temperature (Figure 19a‐2) is one of the intensively studied thin film electrodes.^[^ ^208^, ^209^, ^210^, ^211^, ^212^, ^213^, ^214^, ^215^, ^216^, ^268^ ^]^ The lattice structure of LiCoO_2_ consists of CoO_2_ and Li layers, which are stacked along its c‐axis giving the lattice parameters *a* and *c* to be 2.8138 Å and 14.0516Å, respectively. Due to the stacking along the c‐axis, the Li ions in the layered LiCoO_2_ only exhibit 2D migration pathways along *a*‐ and *b*‐axis. Since high crystallinity is one of the prerequisites for successful intercalation and deintercalation, to ensure a good crystallinity, deposition at high substrate temperature usually higher than 500 °C is essential.

Figure 19b reveals the XRD of the LiCoO_2_ thin film electrode deposited at a substrate temperature of 600 °C showing a sharp (003) diffraction, indicating a good crystallinity. In addition to deposition temperature and/or postannealing temperature, the electrochemical performance of the LiCoO_2_ electrode is also very sensitive to deposition working pressure. At a low deposition working pressure, for example, the sputtered electrode possesses mixed rhombohedral and cubic structures accompanied with a fairly high amount of impurity of Co_3_O_4_, while at high working pressure, the low‐temperature cubic phase is largely reduced together with the reduction of impurity of Co_3_O_4_, monitored by Raman spectra.^[^ ^216^ ^]^ Figure 19c summarizes the effect of the working pressure at a fixed deposition temperature where the working pressure varied from 0.55 up to 3 Pa. The specific capacity of the electrode deposited at 0.55 Pa only shows about 10 mAh cm^−2^ µm^−1^. With an increase in working pressure, the specific capacity increased and reached to the maximum at 3 Pa.^[^ ^216^ ^]^ Preferred orientation of the thin film electrode is another concern for ATFBs. Figure 19d schematically illustrates three typical orientations, which were very often observed in the LiCoO_2_ thin films. Among many different orientations, it is easy to obtain (003) orientation. However, since Li ion migration is along the Li layers, (003) orientation will obviously largely block Li migration pathway. The Li ions can only diffuse through grain boundaries, whereas (101) and (104) orientations expose the Li layers to the electrolyte, hence enhancing Li kinetics. Several parameters may control and affect the orientation of the cathode, including the orientation of the current collector below the cathode film, oxygen partial pressure, and substrate temperature. Figure 19e‐1,2 reveals high resolution TEM of LiCoO_2_ electrodes showing $\left[1\bar{1}0\right]$ and $\left[11\bar{2}\right]$ orientations. The former was deposited onto $\left[1\bar{1}00\right]$ substrate and later onto $\left[11\bar{2}0\right]$ one. To control the film orientation, better understanding of the surface energy and the volume strain energy of the deposited layer is important in order to balance the two energies, namely the surface energy and strain energy of the deposited layer. The surface energy of (003) orientation is the lowest one of about 0.4–1 J m^−2^, whereas the surface energy slightly increases to 1.05 J m^−2^ for (014) orientated film and largely raises to 2.24 J m^−2^ for the film with (110) orientation.^[^ ^271^ ^]^ Therefore, (003) orientation can be obtained naturally. On the other hand, the volume strain energies of the films with orientation (003), (014), and (110) are 126.2, 78.4, and 76.1 J m^−3^, respectively, due mainly to lattice misfit.^[^ ^272^, ^273^ ^]^

**Figure 20:**
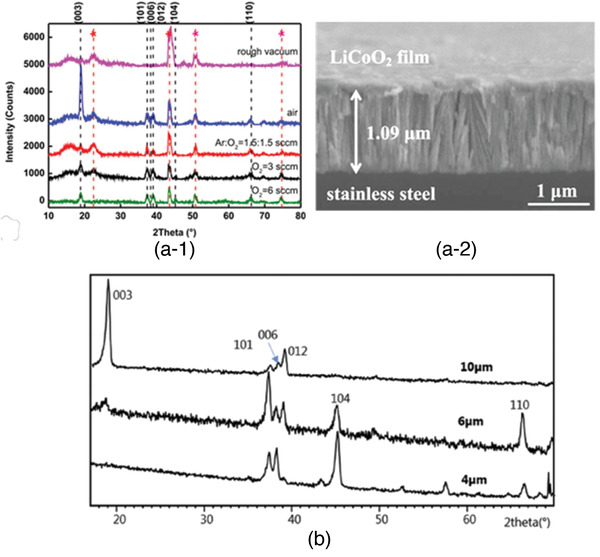
Change of orientation of postannealed LiCoO_2_: a‐1) changes in orientation of postannealed thin films at different atmospheres and a‐2) columnar‐structured thin film after annealing. Reproduced with permission.^[^ ^274^ ^]^ Copyright 2020, Cambridge University Press. b) Dependence of orientation on thickness of deposited film. Reproduced with permission.^[^ ^276^ ^]^ Copyright 2017, Elsevier.

Since the films that are grown at room temperature behave poor crystallinity, postannealing of the films at high temperature is necessary. During the annealing process, atoms of the thin film are rearranged to their lowest energy state through diffusion. Three parameters, namely annealing temperature, duration of annealing and annealing atmosphere, control the crystallinity as well as the orientation of the films. The temperature and duration of annealing mainly govern crystallinity whereas annealing atmosphere not only affects the crystallinity but also strongly influences orientation of the annealed thin films. To achieve a reasonable good crystallinity, the postannealing temperature should be about 600–800 °C which is much higher than substrate temperature at deposition.^[^ ^274^, ^275^, ^276^ ^]^ The reason is that mobility of atoms during deposition is higher since diffusion of atoms is mostly through surface migration leading to lower activation energy. However, crystallization from an amorphous state after low temperature deposition to a crystalline state is through lattice diffusion, which has much high activation energy. As such, higher temperature is required to overcome the high bulk activation energy. Environmental atmosphere during annealing is another important parameter, which also controls orientation of the annealing film since it influences surface energy. **Figure** **20**a‐1 reveals changes of out‐off plan orientation after annealing in vacuum, air, and oxygen environments. A strong (003) orientation appears in the film annealed in air, but it disappears when the film was annealed in oxygen and vacuum condition and forms a columnar structure (Figure 20a‐2). Balance/competition between strain energy and surface energy is reflected by the change of film thickness. For a thin electrode, the strain energy is a dominant factor over the surface energy controlling crystallization orientation. It becomes less dominant when thickness of the film is thick. Figure 20b shows a (104) orientation when the film is as thin as 4 µm. With increase in the thickness of the film to 6 µm, the intensity of (104) orientation is lowed accompanied with formation of (003) diffraction, implying that surface energy becomes dominant. For a 10 µm thick film, (104) orientation almost completely disappears instead of formation of strong (003) diffraction.

A ternary compound such as LiNi*_x_* Mn*_y_* Co*_z_* O_2_, typically with the composition of LiNi_1/3_Mn_1/3_Co_1/3_O_2_, is another type of layer‐structured cathodes, which has the same structure as LiCoO_2_ (*α*‐NaFeO_2_) but behaves better safety and cyclability due to its electronic structure and little cations mixing.^[^ ^218^ ^]^ The film growth temperature or postannealing temperature is about the same as that for LiCoO_2_. The lowest annealing temperature is only 450 °C.^[^ ^219^ ^]^

#### 4.2.2 Spinel‐Structured of Thin Film Electrode

**Figure 21:**
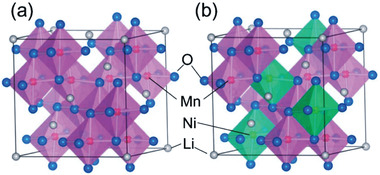
a $Fd\bar{3}m$ space group and b) *P*4_3_32 space group. Reproduced with permission.^[^ ^278^ ^]^ Copyright 2013, The Royal Society of Chemistry.

The chemical composition of a spinel‐structured cathode is LiM_2_O_4_ where M is the transition metal such as V, Ti, Mn, Zn, Ni, Zn. When M = Mn, a classical LiMn_2_O_4_ spinel forms with a basic building block of an octahedron of oxygen atoms with an atom of manganese at its center.^[^ ^277^ ^]^ The octahedral MnO_6_ building blocks are linked by edge‐sharing forming 3D Li^+^ migration tunnel structure as shown in **Figure** **21**a.^[^ ^278^ ^]^ LiMn_2_O_4_ spinel is able to provide one electron from the lowest *e_g_* level with three electrons in the lowest *t_2g_* level.^[^ ^279^ ^]^ The intercalation/deintercalation is through migration of Li^+^ into/from the spinel structure yielding an open circuit voltage of 4.1 V versus Li^+^/Li without change of Mn_2_O_4_ framework. There are two‐step deintercalation and intercalation of Li^+^:

(21) $$\mathrm{LiM}n_{2}O_{4}↔Li_{0.5}Mn_{2}O_{4}+0.5Li^{+}+0.5e^{-}$$

and

(22) $$Li_{0.5}Mn_{2}O_{4}↔\lambda Mn_{2}O_{4}+0.5Li^{+}+0.5e$$

To avoid Jahn‐Teller distortion at deep discharge when the valance state of Mn is low and improve cyclability, Mn is usually partly substituted by transition metals forming LiM*_x_* Mn_2‐_ *_x_* O_4_ where M = Ni, Fe, Co, Cr, Ti, Zn etc. Doping Ni forms LiNi_0.5_M_1.5_O_4_ that has a new potential plateau at 4.8 V.^[^ ^279^, ^280^ ^]^ LiNi_0.5_M_1.5_O_4_ has two space groups, namely $Fd\bar{3}m$ in which the tetrahedral 8a sites are occupied by Li ions while Ni and Mn ions randomly locate in the octahedral 16d sites (Figure 21a), and *P*4_3_32 where Li ions occupy 8c and 4a and 12d sites are orderly occupied by Ni and Mn ions, respectively (Figure 21b).^[^ ^281^ ^]^

Both LiMn_2_O_4_ and LiNi_0.5_M_1.5_O_4_ thin film electrode were fabricated by various technologies including rf‐sputtering,^[^ ^207^ ^]^ PLD,^[^ ^240^ ^]^ electron beam evaporation,^[^ ^228^ ^]^ etc. Compared with layer‐structured LiCoO^[^ ^2^ ^]^ that should be grown at a temperature at least 600 °C, the deposition for LiMn_2_O_4_ is lower.

**Figure 22:**
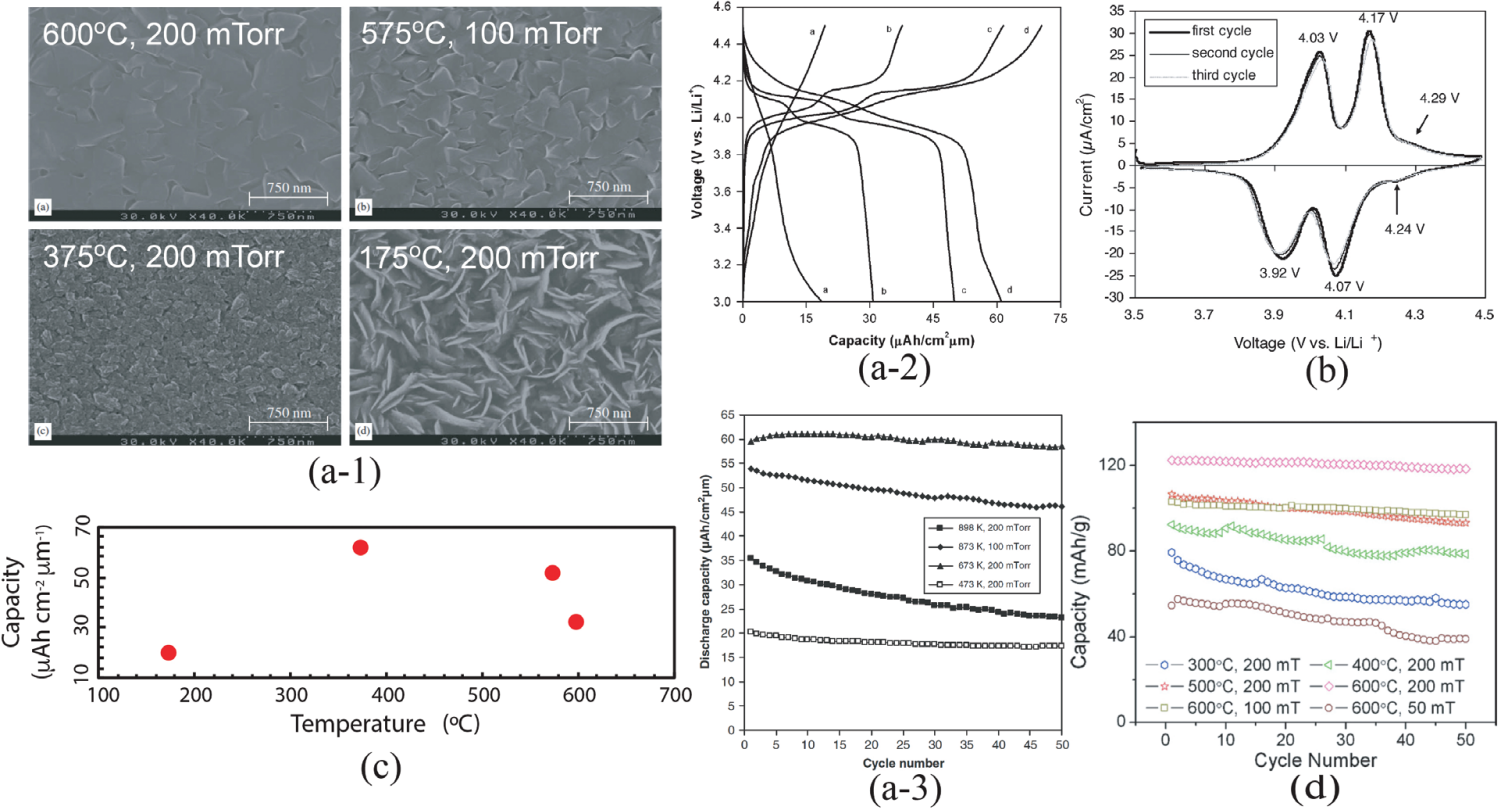
a) Morphology and electrochemical performance of LiMn_2_O_4_: a‐1) Morphologies of thin film electrodes growth at different conditions, a‐2) galvanostatic charge/discharge of the electrodes deposited in the different conditions and a‐3) cyclic performance of different electrodes grown by PLD. Reproduced with permission.^[^ ^282^ ^]^ Copyright 2006, Elsevier. b) Cyclic voltammetry showing two redox reactions. Reproduced with permission.^[^ ^283^ ^]^ Copyright 2006, Elsevier. c) Discharge capacity as a function of substrate temperature, and d) cyclic performance of as a function of deposition temperature and partial pressure. c,d) Reproduced with permission.^[^ ^240^ ^]^ Copyright 2007, Elsevier.

**Figure** **22**a‐1 shows the morphologies of the thin film electrodes deposited at different temperatures and oxygen partial pressure, and clear crystalline features of the thin films grown at 575 and 600 °C can be observed. The XRD also reveals strong (111), (311), (222), and (400) diffractions. When the film was deposited at 375 °C, crystallinity was largely reduced. Only fine plate‐like morphology could be observed when the deposition temperature was lowered to 175 °C in which the intensity of XRD (111) diffraction became extremely low, indicating poor crystallinity. Galvanostatic charge/discharge of the electrodes deposited in the different conditions are depicted in Figure 22a‐2, showing two charge/discharge plateaus being dependent on the deposition temperatures, which can also be reflected by cyclic voltammetry Figure 22b. The capacities of the film labeled as a, b, c, and d correspond to the film deposited at 175, 600, 575, and 375 °C, respectively. The film with a poor crystallinity obviously possessed the lowest discharge capacity of only about 20 µAh cm^−2^ µm^−1^. The highest capacity of 61 µAh cm^−2^ µm^−1^ was achieved when the film was deposited at 370 °C. However, further increase in deposition temperature, the specific capacities were largely reduced (Figure 22c). All four thin‐film electrodes demonstrated equally good cyclability although the specific capacities are different (Figure 22a‐3). Figure 22d reveals variation of capacity of LiNi_0.5_Mn_1.5_O_4_ thin film cathode deposited at different deposition temperature and partial pressure.^[240]^ The films that were grown using rf‐sputtering show the same trend with an optimized deposition temperature of 500 °C.^[284]^ The increase in the specific capacity is associated with increase in crystallinity hence giving rise to vacancy tunning channels for Li^+^ migration. Deposition at a high temperature could lead two negative effects, one of which is interdiffusion between substrate/current collector and the cathode layer causing compositional change along the interface, and another one is Li loss. Therefore, processing at low temperature is desired provided that crystallinity can be ensured.

Substitution of *x* mol Mn with Ni forms LiNi*_x_* Mn_2‐_ *_x_* O_4_ that features a number of advantages over LiMn_2_O_4_, such as minimized Jahn‐Teller distortion and high redox potential. Since the lowest Ni *e_g_* level can hold two electrons, 2*x* electrons can be provided by Ni *e_g_* level. If 2*x* is less than one electron per formula, Mn *e_g_* electron will provide remained ones. Therefore, there are in general three voltage plateaus from $Ni^{2^{+}↔3^{+}}$ and $Ni^{3^{+}↔4^{+}}$, and $Mn^{3^{+}↔4^{+}}$. The former two redox reactions from Ni are at about 4.7 V while the latter at about 4.1 V. Since LiNi_0.5_Mn_1.5_O_4_ has a voltage potential of 4.7 versus Li^+^/Li while the oxidation limit of the current liquid organic electrolytes is only about 4.5 V, LiNi_0.5_Mn_1.5_O_4_ has never been used in the bulk batteries. Since most inorganic electrolytes are stable up to at least 6 V, simply replacement of the relatively low voltage potential LiCoO_2_ by the high voltage potential LiNi_0.5_Mn_1.5_O_4_, for example, the energy density can be increased by 20%. It is particularly important for ASTBs since the thickness of the electrode used in ASTBs is thin, limiting the capacity of the batteries.

**Figure 23:**
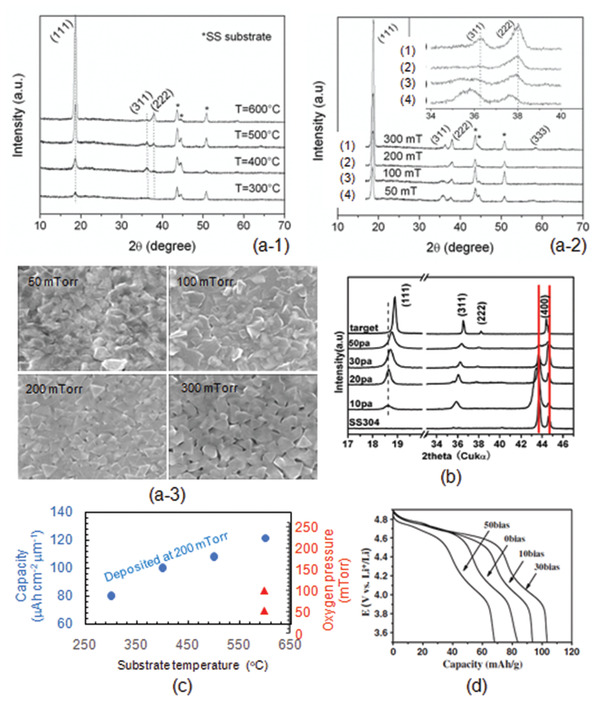
a) Structural evolution and electrochemical performance of LiNi_0.5_Mn_1.5_O_4_: a‐1,2) structural changes as functions of substrate temperature and oxygen partial pressure, a‐3) change of crystallinity and morphology at different oxygen partial pressure. Reproduced with permission.^[^ ^240^ ^]^ Copyright 2007, Elsevier. b) Change of lattice parameter. Reproduced with permission.^[^ ^285^ ^]^ Copyright 2013, Elsevier. c) Specific capacity as a function of substrate temperature and oxygen partial pressure,^[^ ^240^ ^]^ and d) changes of capacity at different bias during deposition. c) Reproduced with permission.^[^ ^240^ ^]^ Copyright 2007, Elsevier. d) Reproduced with permission.^[^ ^249^ ^]^ Copyright 2014, Elsevier.

Substrate and postannealing temperature that is suitable for deposition of Ni substitute LiMn_2_O_4_ is between 600 and 700 °C although crystalline structure appears at the temperature as low as 400 °C.^[^ ^240^, ^285^ ^]^ At 600 °C, the PLD deposited thin film has a good crystallinity (**Figure** **23**a‐1). The crystallinity also changes with oxygen partial pressure at the deposition. The insert in Figure 23a‐2 reveals sharpening of (311) and (222) diffractions with increase in the oxygen partial pressure. The morphologies of the electrodes clearly show the changes from smooth‐edged grains at low oxygen partial pressure of 50 mTorr to sharp‐edged grains at high oxygen partial pressure (Figure 23a‐3). It is worth noting that the oxygen partial pressure not only affects crystallinity but also causes change in composition (Figure 23d).^[^ ^285^ ^]^ At a low oxygen partial pressure, oxygen‐deficient structure, LiNi_0.5_Mn_1.5_O_4‐*δ*_ forms resulting in expansion of the unit cell.^[^ ^285^ ^]^ The specific capacity increases with deposition substrate temperature as well as oxygen partial pressure reaching an optimized oxygen partial pressure 200 mTorr (Figure 23e). The electrode with space group *P*4_3_32 through crystallization at 600 °C also demonstrates the highest capacity, and on the other hand if annealing temperature was too high, only Mn redox reaction could be observed.^[^ ^239^ ^]^ Furthermore, it has also been noted that negative bias employed in deposition would control the size of grains. At no bias, the deposited film shows big grain and the grain size reduces with bias. Due to reduction in grain size, contact area between the electrode and the electrolyte increases as well leading to better ion migration. As a consequence, the specific capacity is enhanced (Figure 23f). However, too high bias causes severe ion bombardment damaging crystallinity.^[^ ^249^ ^]^

#### 4.2.3 Olivine‐Structured of Thin Film Electrode

**Figure 24:**
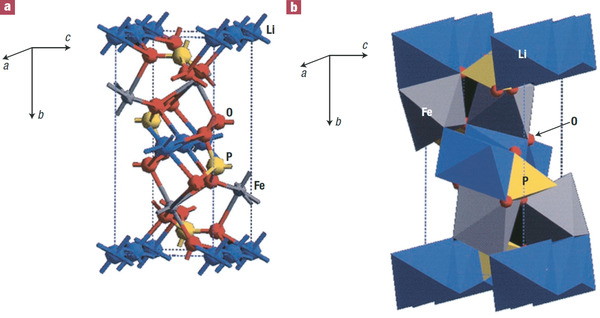
Olivine structure. Reproduced with permission.^[^ ^289^ ^]^ Copyright 2002, Springer Nature.

The compounds LiMPO_4_ where M refers the transition metals including Fe, Mn, and Co have an olivine structure with space group *Pnma*.^[^ ^286^, ^287^ ^]^ Its application in energy storage started from 1997.^[^ ^288^ ^]^ In the olivine structure, the MO_6_ octahedra are corner‐shared, and PO_4_ tetrahedron is edge shared with the MO_6_ octahedron. The cations, M, reside in the M2 sites whereas the cations, Li^+^ occupy M1 sites forming edge‐sharing chains of octahedra in the [100] direction as shown in **Figure** **24**.^[^ ^289^ ^]^

The discharge voltages for M = Fe, Mn, Co, Ni are 3.4, 4, 4.8, and 5V, respectively. LiFePO_4_ is the most well‐studied cathode. Its deintercalation will lead to a theoretical specific capacity of 170 mAh g^−1^. However, its electronic conductivity is as low as 10^−10^ to 10^−9^ S cm^−1^ due to its polyanion nature.^[^ ^289^, ^290^ ^]^

During deintercalation, Li^+^ is extracted from the olivine LiFePO_4_ by formation of MPO_4_, and via versa as showing in Equation (22)

(23) $$\mathrm{LiFeP}O_{4}↔\left(1-x\right)\mathrm{LiFeP}O_{4}+xLi^{+}+xe^{-}+\mathrm{FeP}O_{4}$$

**Figure 25:**
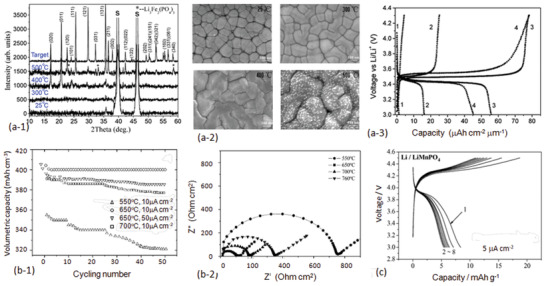
a) Structure and electrochemical performance of olivine‐structured cathode thin‐film electrodes: a‐1) XRD spectra of LiFePO_4_ deposited at different substrate temperatures, a‐2) morphologies of LiFePO_4_ thin film electrodes deposited at different temperatures, and a‐3) galvanostatic charge/discharge plot where 1, 2, 3, and 4 correspond to substrate temperature 25, 300, 400, and 500 °C, respectively. Reproduced with permission.^[^ ^256^ ^]^ Copyright 2009, American Chemical Society. b) Cyclability and electrochemical impedance spectra: b‐1) cyclability of LiFePO_4_ crystallized between 550 and 700 °C and b‐2) impedance spectra of LiFePO_4_ crystallized between 550 and 760 °C. Reproduced with permission.^[^ ^293^ ^]^ Copyright 2014, Royal Society of Chemistry. c) Galvanostatic charge/discharge plot of LiMnPO_4_ crystallized at 600 °C. Reproduced with permission.^[^ ^294^ ^]^ Copyright 2005, Elsevier.

Deposition temperature for LiFePO_4_ ranges from as low as 400 °C to 600 °C whereas the postannealing temperature is carried out between 500 and 750 °C, typically at about 500 °C in a protective or reducing gas environment such as Ar, N_2_, Ar+H_2_, N_2_+H_2_.^[^ ^255^, ^291^, ^292^ ^]^ **Figure** **25**a‐1 shows the XRD spectra of the LiFePO_4_ thin film electrodes that were rf‐sputtered from the room temperature to 500 °C.^[^ ^256^ ^]^ The olivine structure is clearly observed when the deposition was conducted at the substrate temperature of 400 °C. Their corresponding morphologies are given in Figure 25a‐2.^[^ ^256^ ^]^ The grain size increases with increase in deposition temperature. There are a lot of small particles of about a few nanometers which is most likely Li_3_Fe_2_(PO_4_)_3_ impurity when the film was deposited at 500 °C. Significant difference in the specific capacity is depicted in Figure 25a‐3.^[^ ^256^ ^]^ The film deposited at room temperature shows almost no capacity due to its amorphous structure and the specific discharge capacity increases with deposition temperature and peaks at 400 °C (line 3 in Figure 25a‐3). The specific capacity reduced to 45 µAh cm^−2^ µm^−1^ when the film was deposited at 500 °C. The reduction of the discharge capacity can be attributed to formation of the impurity phase of Li_3_Fe_2_(PO_4_)_3_. Compared with oxide cathodes such as layer‐structured and spinel‐structured thin films, the deposition temperature for olivine‐structured LiFePO_4_ seems lower. The LiFePO_4_ film electrode through postannealing shows the same trend. As shown in Figure 25b‐1, the highest specific capacity and best cyclability could be achieved for the film which was postannealed at 650 °C. Further increase in the postannealing temperature led to reduction in specific capacity.^[^ ^293^ ^]^ Figure 25b‐2 shows Nyquist plots of the thin film electrodes after 50 charge/discharge cycles. Very high impedance of 750 Ω cm^2^ was noted for the film that was annealed at 550 °C due to poor crystallinity and the impedance was sharply reduced to about 65 Ω cm^2^ for the film that was annealed at 650 °C but was increased if annealing temperature was too high. The film annealed at 760 °C was increased to almost 300 Ω cm^2^ suggesting structural and compositional change leading to reduced Li^+^ migration.

Like LiFePO_4_, LiMnPO_4_ is also polyanion olivine‐structured cathode that possesses higher voltage plateau of 4 V versus Li^+^/Li. During deintercalation, LiMnPO_4_ transforms to MnPO_4_, and the two phases have large lattice mismatch. As such, Li^+^ migration needs to overcome a high energy barrier for Li^+^ to across the phase boundary. In addition, strong localization of electrons and holes around the centers of Mn also causes polaron hopping between adjacent metal centers to be extremely difficult.^[^ ^296^, ^297^, ^298^ ^]^ Therefore bare LiMnPO_4_ has very low electronic conductivity. Due to poor electronic conductivity, the LiMnPO_4_ film shows very low specific capacity (Figure 25c).^[^ ^294^ ^]^

**Figure 26:**
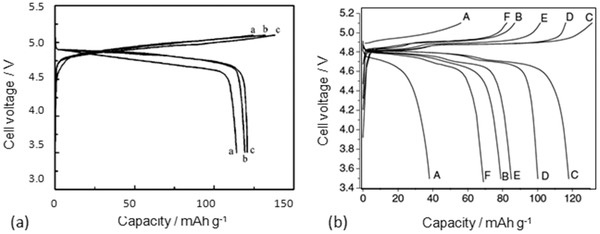
Galvanostatic measurement of Li_1+_ *_x_* CoPO4: a) LiCoP_4_ in different conditions (Reproduced with permission.^[^ ^295^ ^]^ Copyright 2004, IOP Publishing), and b) Li_1+_ *_x_* CoPO_4_ where *x* = 1, 0.2, 0.4, 0.6, 0.8, and 1.0 corresponding to charge/discharge lines A, B, C, D, E, F. Reproduced with permission.^[^ ^300^ ^]^ Copyright 2006, Elsevier.

Different from LiFePO_4_ and LiMePO_4_, deintercalation of Li^+^ from LiCoPO_4_ takes place through two stages, with an intermediate phase of Li_0.67_CoPO_4_ together with fully lithiated LiCoPO_4_.^[^ ^299^ ^]^ There are limited articles on LiCoPO_4_ thin film electrode since most of the thin film electrode studies are still based on a liquid organic electrolyte that only possesses a narrow stable potential window up to 4.5 V. Although the LiCoPO_4_ thin film electrode showed a quick fade due to electrolyte oxidation, it still demonstrated a high capacity of about 120 mAh g^−1^ and little polarization. **Figure** **26**a reveals the charge/discharge capacity of LiCoPO_4_ that was sputtered and postannealed at 400 °C in a: LiPF_6_ and b: LiBF_4_ electrolytes, and c: Al_2_O_3_‐modified LiCoPO_4_ in LiPF_6_ electrolyte.^[^ ^295^ ^]^ Comparing the discharge capacity a and b in Figure 26a, the Al_2_O_3_‐modified LiCoPO_4_ demonstrated better stability in LiPF_6_ electrolyte. Annealing in protective environment for polyanion electrode is necessary. As an example, the effect of excess of Li in LiCoPO_4_ is given Figure 26b in which the excess Li ranges from 0.2 to 1.0 per formula. For the stoichiometric composition, the discharge capacity (line A) is the lowest one. After introducing 0.4 excess Li salt, the highest capacity of about 118 mAh g^−2^ with the lowest impedance could be obtained.^[^ ^300^ ^]^

## 5 Technologies for Thin‐Film μ‐Batteries

There are many available deposition technologies for thin film growth, including sol‐gel method,^[^ ^301^, ^302^, ^303^, ^304^ ^]^ inkjet printing,^[^ ^305^, ^306^ ^]^ rf‐sputtering,^[^ ^174^, ^206^, ^210^, ^229^, ^230^, ^258^, ^307^, ^308^ ^]^ plasma spray PVD (PS‐PVD),^[^ ^309^ ^]^ PLD,^[^ ^310^, ^311^, ^312^ ^]^ chemical vapor deposition (CVD),^[^ ^313^ ^]^ aerosol deposition,^[^ ^314^ ^]^ ion‐beam assisted deposition,^[^ ^315^ ^]^ and e‐beam evaporation.^[^ ^316^ ^]^ The review article by Lobe et al.^[^ ^308^ ^]^ well documented various deposition technologies. Among many different types of deposition technology, rf‐sputtering is the most mature one that has been widely used from the laboratory scale to industrial scale. The rf‐sputtering is able to deposit well‐controllable composition and thickness through adjusting sputtering power, working pressure, duration of sputtering, and even bias. The sputtering machine with single target and multiple targets cosputtering is available. Another advantage of the use of the rf‐sputtering is in situ nitriding such as the growth of LiPON.^[^ ^317^, ^318^, ^319^, ^320^ ^]^

**Figure 27:**
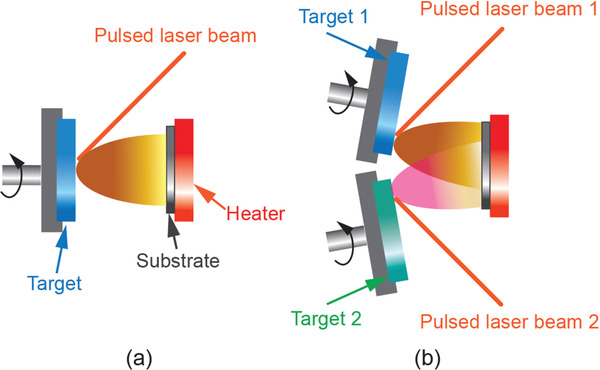
Schematic illustration of the principle of PLD: a) using a single target, and b) dual targets used for compositional studies.

PLD is a versatile technique for laboratory application of thin‐film growth. PLS uses a pulsed laser beam to irradiate a target in **Figure** **27**a. The laser irradiation causes formation of a plum from the target. Usually the plum contains atoms, ions, and even droplets depending on the composition and quality of the targets, and wavelength and energy density of the laser etc. Use of two targets was also proposed where the two targets have different compositions and the two plums generated from the two targets eject species with different compositions to the substrate (Figure 27b). Since the substrates are stationary, on one edge of the substrate the composition will be very different from another edge. By using this method, continuous compositional changes can be achieved just from one deposition. Similar to the rf‐sputtering, to obtain a crystalline, the substrate must be heated to a high temperature. Compared to rf‐sputtering, uniformity of chemical composition of PLD‐ablated thin film is poor due to presence of atoms and droplets during ablation. The article of pulsed laser deposition as a tool for the development of all solid‐state microbatteries well summarized various contributions towards qualities of thin‐film batteries.^[^ ^312^ ^]^

**Figure 28:**
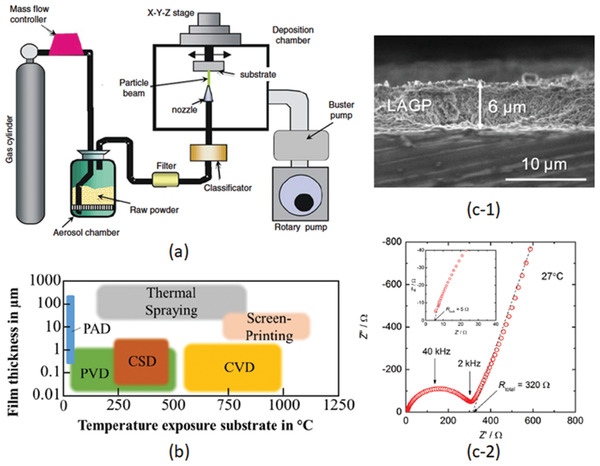
Processing of ASTBs through AD: a) illustration of mechanisms of AD. Reproduced with permission.^[^ ^322^ ^]^ Copyright 2008, Springer Nature. b) Comparison of various deposition technologies. Reproduced with permission.^[^ ^323^ ^]^ Copyright 2019, World Scientific Publishing Co., Inc. c‐1) SEM image of ADed LAGP electrolyte and c‐2) impedance of the ADed LAGP. Reproduced with permission.^[^ ^324^ ^]^ Copyright 2015, Elsevier.

One of the technologies named as aerosol deposition (AD) and also called powder aerosol deposition (PAD) is worth mentioning. **Figure** **28**a schematically illustrates the deposition system.^[^ ^321^, ^322^ ^]^ The whole system consists of a deposition chamber, an aerosol chamber (container), a nozzle, and a number of other attachments. Submicron‐sized powder particles in the aerosol form are subjected to a high pressure, flowing out of the nozzle with a ultrasonic speed impacting onto the substrate (solid electrolytes etc). The powder with high kinetics results in heavy deformation, crushing, and cold‐welding. As a consequence, a film forms. Figure 28b compares different deposition technologies. The AD with much thick films and much fast deposition rate can be achieved at room temperature.^[^ ^323^ ^]^ Figure 28c‐1 reveals one of the examples of fabrication of ASTBs where an LAGP solid electrolyte was deposited onto a substrate. The aerosol deposited (ADed) film has a low impedance (Figure 28c‐2).^[^ ^324^ ^]^ It should be noted that crystallinity will somewhat be reduced after AD because of heavy deformation and crushing of the powder particles.

One of the obvious advantages of the AD is its capability to deposition of crystalline films at room temperature. Since the interdiffusion between different components can be avoided when it is operated at the room temperature, the powder with multicompositions can be codeposited. For example, the electrode powder can be premixed with a certain amount of solid electrolyte and electron conductive material and then they are codeposited creating electrode layer with both ions and electrons pathways.

## 6 Challenges and Prospects

Although the ASTBs find their practice applications in microelectronics and miniature devices, they are still facing a number of challenges.

### 6.1 Enhancement of Capacity and Energy

**Figure 29:**
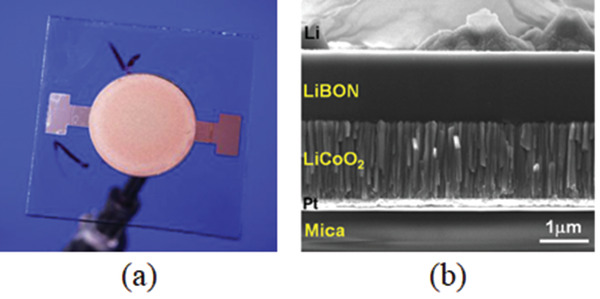
Planar thin film: a) image of 2D thin‐film battery. Reproduced with permission.^[^ ^325^ ^]^ Copyright 2016, Elsevier. b) Cross sectional image. Reproduced with permission.^[^ ^326^ ^]^ Copyright 2016, Elsevier.

The ASTBs in the planer format have been successfully investigated. Usually, the thickness of electrodes is as thin as a few micrometers or less, because poor electronic and ionic conductivities limit the thickness of the electrode thin film. **Figure** **29**a shows the example of an ASTB in a planar format, and Figure 29b is the cross‐sectional view of another example where the thickness of the cathode electrode is less than 2 µm.^[^ ^325^, ^326^ ^]^ Because of constraining in the thickness of the cathode electrodes, the capacity of the planar ASTBs is dependent on the deposition area of the ASTBs since it is not possible to adopt thick electrode. Therefore, increase in the deposition area becomes the key to increase capacity, leading to some excellent designs.^[^ ^20^ ^]^

**Figure 30:**
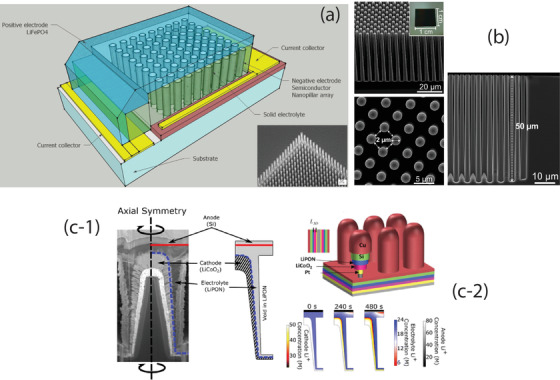
3D ASTBs: a) concept of 3D μ‐battery. Reproduced with permission.^[^ ^329^ ^]^ Copyright 2011, Elsevier. b) Enlarged 3D pillar arranges. Reproduced with permission.^[^ ^330^ ^]^ Copyright 2017, American Chemical Society. c) Microstructure and illustration of 3D μ‐battery. Reproduced with permission.^[^ ^332^ ^]^ Copyright 2020, AIP Publishing.

Since surface area of a substrate is limited, 3D structure becomes the most important design concept because it can increase the deposition area within the limited constrain surface.^[^ ^20^, ^327^, ^328^ ^]^ **Figure** **30**a schematically illustrates the concept of a 3D μ‐battery which consists of micropillar arrays.^[^ ^329^ ^]^ The insert in Figure 30a shows an SEM image of the micropillar arrays. The enlarged micropillar arrays with 2 µm in diameter and 50 µm in height are depicted in Figure 30b. The interspacing of 2 µm between different pillars is for deposition of the electrode materials and the electrolyte.^[^ ^330^, ^331^ ^]^ Cross‐sectional TEM image depicted in Figure 30c‐1 reveals proof‐concept of the 3D μ‐battery pillar with its illustration in Figure 30c‐2.^[^ ^332^ ^]^ The pillar acts as a support of “one” μ‐battery cell that was deposited with the current collector, Pt, as the first layer and the current collector is connected with the rest current collectors on the other pillars forming numerous μ‐battery connected in parallel. On top of the current collector, a cathode layer, LiCoO_2_ in this example, was deposited followed by LiPON as the electrolyte and Si as the anode. Finally, negative current collector, Cu, was deposited forming a completed single μ‐battery cell. Depending on the length of the pillar, the capacity of the ASTB can be enhanced over 10th times.

Use of the cathode with high voltage potential, such as LiCoPO_4_, LiNiPO_4_, and LiNi_0.5_Mn_1.5_O_4_ is another way to increase the energy. These cathodes that are unable to be used with liquid organic electrolytes can be utilized with the solid electrolytes.

### 6.2 Low Temperature Deposition

ASTBs have been fabricated through various deposition technologies, including rf‐sputtering,^[^ ^174^, ^206^, ^210^, ^229^, ^230^, ^258^ ^]^ PLD,^[^ ^251^, ^333^ ^]^ and chemical solution methods,^[^ ^234^, ^334^ ^]^ etc. among which rf‐sputtering is widely used since it is a very mature process and capable to fabricate the thin film electrolytes and electrodes with various composition and can be used for large‐scale fabrication as well. Regardless of which type of deposition technologies mentioned above, except for AD, deposition of the cathodes or postannealing at high substrate temperature is necessary in order to achieve good crystallinity. Therefore, the cathode layers are usually deposited as the first layer immediately after current collector if a suitable current collector is selected.

**Figure 31:**
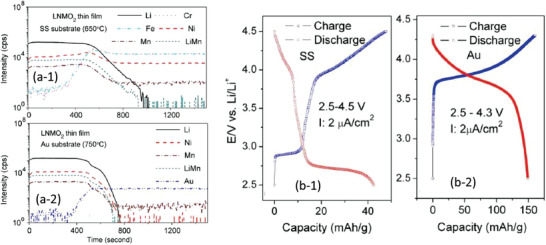
Influence current collector on the composition of electrode and electrochemical performance: a‐1) depth profile of composition of the film on stainless steel current collector, a‐2) depth profile of composition of the film on Au current collector, b‐1) galvanostatic plot of the electrode on stainless steel current collector, and b‐2) galvanostatic plot of the electrode on Au current collector. Reproduced with permission.^[^ ^335^ ^]^ Copyright 2008, AIP Publishing.

**Figure** **31** shows the influence of current collector on the compositional depth profile and electrochemical performance of the LiNi_0.5_Mn_1.5_O_4_ electrode film. Two different current collectors, namely stainless steel and Au were used. Although deposition of the LiNi_0.5_Mn_1.5_O_4_ electrode film on Au was at 750, the interlayer diffusion is as small as a half of the one on the stainless steel (Figure 31a‐1,2) since Au is much stable in comparison to stainless steels. Due to compositional change, the electrode prepared on the stainless steel has low voltage potential and capacity.^[^ ^335^ ^]^

Deposition of following layers, such as electrolyte and anode, should be carried out at low temperatures to avoid interdiffusion. Although rf‐sputtering, PLD, CVD, sol‐gel deposition technologies can be used for ASTB processing, the films with a single composition can only be produced. As discussion in the section of deposition, most electrode materials have poor ionic as well as poor electronic conductivity, which is not an issue for bulk batteries since conductivity carbon and liquid electrolyte used in the bulk batteries are used to facilitate the electrons’ and ions’ migration. Although doping can engineer electronic as well as ionic conductivity of electrode materials, the limited improvement is still far from satisfactory. Therefore, the incorporation of electronic and ionic conductive materials into electrode becomes a possible solution. As discussed in the Section 5, the AD method may be a good consideration of choices since multi‐compositions can be deposited at the sometimes without interdiffusion.

### 6.3 Reduction of Internal Strains

**Figure 32:**
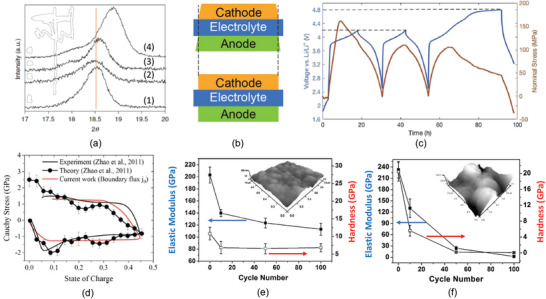
Variation of stresses and effects: a) change of lattice parameter of the electrode during delithiation in which (1), (2), (3), and (4) represent diffraction spectrum of the as‐deposited film with an OCV 3 V, that after 300 cycles at 3 V, that after delithiation to 4.05 V, and that after delithiation to 4.5 V, respectively, b) schematic illustration of volume change, c) stress changes during lithiation and delithiation. a‐c) Reproduced with permission.^[^ ^339^ ^]^ Copyright 2017, Elsevier. d) Experimentally measured stress and theoretical calculation. Reproduced with permission.^[^ ^340^ ^]^ Copyright 2019, Elsevier. e) Change of mechanical responses of cycled LiMn_2_O_4_. Reproduced with permission.^[^ ^341^ ^]^ Copyright 2012, Elsevier. f) Change of mechanical responses of cycled RuO_2_. Reproduced with permission.^[^ ^342^ ^]^ Copyright 2013, Springer Nature.

Generation of internal strain and hence stress is another challenge but has been less studied. Only in recent years, it has been gradually received more attentions.^[^ ^336^, ^337^, ^338^ ^]^ The internal strain in each component of the ASTBs can be caused by at least two parameters, namely change in electrochemical state and temperature. Compared to electrochemical state, thermally induced strain is negligible. **Figure** **32**a depicts the change in lattice parameter of a positive electrode from the observation of the XRD spectra. Diffraction spectrum 1) is the diffraction of the as‐deposited cathode with an open‐circuit voltage (OCV) 3 V. There was almost no change in diffraction angle after 100 galvanostatic cycles but the peak was broadened indicating a decrease in crystallinity (spectrum (2)). Spectrum (3) and (4) represent the diffractions when the film was delithiated to 4.05 V and 4.5 V, respectively. A slight reduction in the lattice parameter was observed after delithiation to 4.05 V and large change at 4.5 V. Since theoretically there should be no volume change of the electrolyte during galvanostatic cycling, the volume shrinkage during delithiation causes in plane tensile stress as illustrated in Figure 32b. Stress measurement shows an increase in the nominal in plane stress of the cathode (Figure 32c) with the degree of delithiation followed by a reduction during lithiation.^[^ ^339^ ^]^ The change in stress of Si anode given in Figure 32d shows the same trend.^[^ ^340^ ^]^ Due to the restriction of the in plane strain by the electrolyte, shear stresses between the electrodes and the electrolytes are therefore generated. The repeated in plane normal stresses and shear stresses will result in cracks and delamination if the stress level exceeds their mechanical properties. Figure 32e shows changes of elastic modulus and hardness of LiMn_2_O_4_ as a function of charge/discharge cycles.^[^ ^341^ ^]^ The modulus as well as hardness of the as‐deposited cathode quickly dropped even after ten cycles, indicating a weakening of adhesion between the cathode and the electrolyte, and of bonding between different grains. Figure 32f reveals the same measurement but on conversion type of electrode material, RuO_2_, in which elastic modulus and hardness continuously reduced. The hardness is almost zero after 100 cycles.^[^ ^342^ ^]^ It is also noted from the inserts of the two figures, relatively small change in roughness of the cathode surface but extraordinarily large change for conversion type of material. These observations indicate importance of selection of different types of electrode materials. To reduce the in plane strain, the materials, such as polyanion type cathodes and spinel Li_4_Ti_5_O_12_ etc. with little volume deformation during galvanostatic cycling are preferred.

### 6.4 Interfaces

Interface is another big challenge. Since it is associated with physicochemical and electrochemical stability of different components, monitoring changes along the interface between two different components is extremely difficult. Many studies in which some of them were based on impedance measurement have shown good physical stability of the interface between LiPON and Li metal while others noted formation of Li_3_PO_4_, Li_3_P, Li_3_N, and Li_2_O at the anode electrolyte interface.^[^ ^39^, ^180^ ^]^ X‐ray photoemission measurement showed the decrease of the O_b_ component respective to O_nb_, indicating that the Li metal reacted with the bridging oxygen.^[^ ^40^ ^]^ In situ TEM directly observed immediate expansion of LiPON toward Li metal side when it was in contact with a Li metal source.^[^ ^343^ ^]^ Although there is interfacial reaction between Li and LiPON, Schwobel et al.^[^ ^40^ ^]^ concluded “the interface reactions are not continuous, but quickly vanish due to the formation of a suitable passivation layer, meaning that the chemical reactions are finished after a certain period of time and not all the LiPON is destroyed.” The first principle calculation by Zhu et al.^[^ ^344^ ^]^ also revealed instability of Li metal against LiPON due to a narrow electrochemical window with a reduction potential of 0.69V versus Li/Li^+^. Nerveless the formation of the interface will cause increase in impedance and hence voltage polarization. In order to overcome the formation of a thick interfacial layer, a ultrathin artificial interfacial layer between the Li metal and the electrolyte is created. Xiao et al. deposited Al_2_O_3_ layer of about a nanometer in thickness to prevent interaction between the anode and the electrolyte.^[^ ^180^ ^]^ Since Al_2_O_3_ is stable, this battery demonstrated a long‐term cycling and ultrafast rate capability. Cathode electrolyte interface formed between the cathode and the solid electrolyte seems stable at room temperature, however, at relatively high temperature the thickness of the cathode electrolyte interface quickly grows. One of the examples is the observation of the cathode electrolyte interface between LiPON and lithium cobalt oxide (LCO). The thickness of the cathode electrolyte interface remained unchanged if the battery was electrochemically cycled at 25 °C while the thickness of the cathode electrolyte interface gradually increased to about 3–4 µm after 250 galvanostatic cycles at only 80 °C.^[^ ^345^ ^]^ More importantly, the thickness change rate of the cathode electrolyte interface is state of charge dependent even in the static state.^[^ ^345^ ^]^ These issues must be properly addressed in the design the format of the ASTBs.

Although there are limited applications of the ASTBs at this stage, with the discovery of new materials and processing which is compatible with CMOS processing, the ASTBs are expected to have wide applications for further miniature of microelectronic devices and nano‐electromechanical systems.

## Conflict of Interest

The authors declare no conflict of interest.
